# Cancer mortality in the Capitals and in the interior of Brazil: a four-decade analysis

**DOI:** 10.11606/s1518-8787.2020054002255

**Published:** 2020-11-23

**Authors:** Gulnar Azevedo e Silva, Beatriz Cordeiro Jardim, Vanessa de Melo Ferreira, Washington Leite Junger, Vania Reis Girianelli

**Affiliations:** I Universidade do Estado do Rio de Janeiro Instituto de Medicina Social Departamento de Epidemiologia Rio de JaneiroRJ Brasil Universidade do Estado do Rio de Janeiro. Instituto de Medicina Social. Departamento de Epidemiologia. Rio de Janeiro, RJ, Brasil; II Instituto Nacional de Câncer José Alencar Gomes da Silva Rio de JaneiroRJ Brasil Instituto Nacional de Câncer José Alencar Gomes da Silva. Rio de Janeiro, RJ, Brasil; III Fundação Oswaldo Cruz Escola Nacional de Saúde Pública Sérgio Arouca Rio de JaneiroRJ Brasil Fundação Oswaldo Cruz. Escola Nacional de Saúde Pública Sérgio Arouca. Rio de Janeiro, RJ, Brasil

**Keywords:** Neoplasms, epidemiology, Mortality, trends, Time Series Studies, Health Status Disparities

## Abstract

**OBJECTIVE:**

to describe the trend of mortality from general cancer and more frequent types among men and women living in the Capitals and other municipalities of the five macro-regions of Brazil between 1978 and 2017.

**METHODS:**

Time series study with mortality data corrected by redistribution of ill-defined causes. Proportional cancer mortality was calculated for Brazil and regions. The annual percentage change in rates for total cancer and specific types in each segment and in the selected unit of analysis was calculated by generalized linear regression with Gaussian binding.

**RESULTS:**

the proportion of cancer increased progressively for both sexes from 1978 to 2017. Important differences between the Capitals and the interior of the macro-regions were seen with disaggregated data. The greatest declines occurred for stomach cancer, except in the northern and interior regions of the Northeast, and for the cervix cancer, with a generalized fall, with the exception of the interior of the northern region. Lung cancer decreased among men in the Southeast and South regions and had a generalized increase among women. Breast and prostate cancers tended to decrease in the Southeast and South regions and among residents of the Capitals but showing an increase in the interior of the North and Northeast regions. Colorectal cancer had a general tendency to increase; with stability among men in the Capitals of the South region and among women of the Southeast and Midwest regions and, since 2007, a decrease among women in the South region.

**CONCLUSIONS:**

Cancer mortality showed great variation among residents of capitals and the interior of the country's major regions. Clear decrease in mortality was seen for the main types in the Southeast and South regions. The North and Northeast regions have patterns compatible with cancers associated with poverty, while the large increase of the cancers related to sedentary lifestyle stand out.

## INTRODUCTION

Cancer is currently the second leading cause of illness and death in the world, with wide variation in incidence and mortality among countries due to socioeconomic status and exposure to risk factors related to social conditions and lifestyle.^1^ The occurrence of cancer in the world has increased regardless of demographic growth; in 2012, an estimated 14.1 million new cases and 8.2 million deaths were estimated, and a more significant growth in the worldwide incidence of the disease is expected over the next 20 years ^2^. In developed countries, although there is still an increase in the incidence of some types of cancer, mortality has shown a significant decrease in the last two decades^3^.

The analysis of current trends in cancer indicates that the ongoing economic and social transitions in several middle- and low-income countries are related to increased exposure to reproductive and hormonal risk factors and changes in food standards^4^. Monitoring the magnitude of the incidence and mortality from cancer in a population is a fundamental element for defining priorities and actions directed to its control, as well as for evaluating the effectiveness of the interventions instituted.

In Brazil, cancer mortality in the early 1980s still showed a typical pattern of middle- and low-income countries, with significant rates of cervical and stomach cancers. A transition, however, was already outlined, with an increase in the types associated with the best socioeconomic conditions (breast, prostate and colorectal)^5^ marked by differentiated patterns among the macro-regions of the country and among residents of the Capitals and other municipalities.

Although cancer trends have been reported in Brazil and Federative units (FU) between 1990 and 2015^6^, the existing differences in relation to the place of residence, comparing the data of inhabitants of the Capitals with those of the interior in the five major regions of the country, have not yet been explored in longer series. Knowing the evolution of cancer as a function of the changes that have been occurring in the specific risk patterns and the possibilities of access to treatment is fundamental for the improvement of regionalized control policies.

In addition, it should be considered that the *Sistema de Informações sobre Mortalidade* (SIM – Mortality Information System) was implemented in Brazil by the Ministry of Health (MH) in the late 1970s, with the death declaration as a source document. Since then, MH has been working to improve its completeness and coverage^7^. The series of consolidated data available by SIM until 2017 allows the construction of extended historical series, which is advisable for monitoring the various types of cancer.

This study aims to describe the four-decade trend (1978-2017) of mortality of all cancers and the most frequent types among men and women living in the Capitals and other municipalities of the macro-regions of Brazil.

## METHODS

This is a time series study that had as source the SIM data referring to the years between 1978^8^ and 2017^9^. For the year 1978, the eighth codification of the Manual of the International Statistical Classification of Diseases Injuries and causes of death (ICD-8) was used, between 1979 and 1995, the ninth (ICD-9), and between 1996 and 2017, the tenth (ICD-10).

The population denominators were those of the Brazilian Institute of Geography and Statistics (IBGE) censuses for the years 1980, 1991, 2000 and 2010^9^. For the intercensitary years, linear interpolation was performed. For the later period (2011 to 2017), the population projections were extrapolated also linearly, with disaggregation by sex, age group and place of residence (FU and Capitals) based on the previous period.

In men, the main types were: lung (ICD-9: 162; ICD-10: C33-34), prostate (ICD9: 185; ICD10: C61) stomach (ICD9: 151; ICD10: C16), colorectal (ICD-9: 153-154.1; ICD-10: C18-20), and in women were: breast (ICD-9: 170; ICD-10: C50), lung (ICD-9: 162; ICD-10: C33-34), colorectal (ICD-9: 153-154.1; ICD-10: C18-20), and the cervix (ICD-9: 180; ICD-10: L54). Deaths that had ignored information for sex, age and municipality of residence were excluded. As for the year 1978, only proportional mortality from cancer was calculated, the ICD codes referring to the set of cancers (ICD-8: 140-209) and the chapter referring to the ill-defined causes (ICD-8: Ch. 16).

The correction of the information on the underlying cause of death was done according to the methodology proposed by Mathers et al.^10^ and the adaptation made by Girianelli et al.^11^. This adaptation, which consists in proportionally redistributing 50% of deaths with a basic cause classified as undefined (ICD-8: Ch. 16, ICD-9: 780-799; ICD – 10: R00-R99), is necessary because cancer is less found among ill-defined deaths than among well-defined ones^12^. Thus, the correction factors were calculated for each age group (0-4, 5-14,15-19, 20-29, 30-49, 50-59, 60-69, 70-79 and 80 years or more), calendar year, sex and place of residence (capitals and other municipalities) of the five macro-regions of the country. For deaths recorded as cervical cancer, an additional correction was made with redistribution of all deaths classified as malignant neoplasm of the uterus without other specification (ICD-9: 179; ICD-10: C55), maintaining the proportion recorded as deaths from cervical and uterine body cancer^13^.

With corrected data, the proportional mortality from cancer was calculated for Brazil. Since the 1978 data did not cover all regions, the proportional mortality series for macro-regions was started in 1979.

Then, crude and age-standardized cancer mortality rates were calculated(0-4, 5-14,15-19, 20-29, 30-49, 50-59, 60-69, 70-79 and 80 years or more) and for each sex by the direct method, taking as standard population the world population proposed by Segi^14^ for the 1980- 2017 period. These rates were calculated in a disaggregated manner by macro-regions, capitals and other municipalities (interior).

To estimate the trend, a linear regression model was used. For series with structural breaks, the time variable was introduced into the model by means of linear *spline* by parts, with break points in the years in which the trend changed its trajectory. The identification of the break points was visual and cubic *splines* with 10 degrees of freedom were used to highlight the points of inflection of the curve, allowing to define break points at specific moments of time and the respective trend in each interval, adjusting by a continuous line segment between the points. This approach was chosen because it concerns time series with trends that vary in time in a non-regular way, which makes inappropriate a linear model for global trend for the entire period.

The models were evaluated via residues to verify if the usual assumptions were met, in particular the existence of residual autocorrelation through the functions of autocorrelation (FAC) and partial autocorrelation (FPAC). Models that showed significant autocorrelation in residues or with an absolute value greater than 0.5 were re-estimated using generalized least squares with a first-order autoregressive model AR(1) to model the autocorrelation and correct the variance of the coefficient estimators. We used the function “gls” with estimation for restricted maximum likelihood (REML) of the package “nlme”^15^.

The coefficient of the term for each segment expresses the logarithm of the trend in that interval. Thus, the annual percentage change (APC) of mortality rates was calculated by the formula and respective p values.

For the interpretation of trends, statistical non-significance (p-value above 0.05) was used as a criterion to characterize an APC as stable. The statistically significant APC, when positive, indicated an increasing trend and, when negative, a decreasing trend. The analyses were performed in the R program, version 3.4.1.

## RESULTS

**Figure 1:**
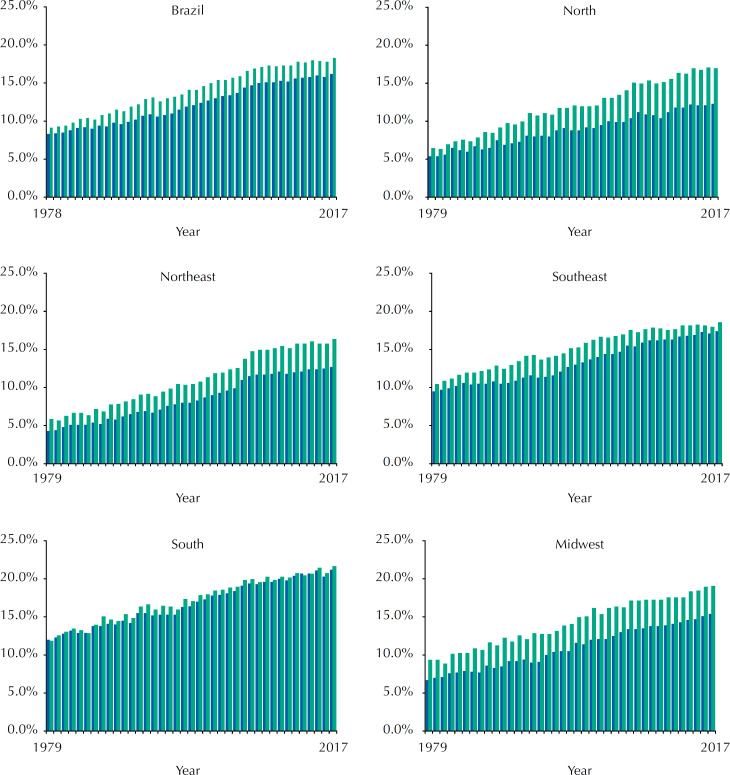
Proportional mortality from cancer, all types, in men and women in Brazil and macro-regions, 1978 to 2017.

Proportional mortality from cancer increased from 1978 to 2017 in men, from 8.3% to 16.2%, and in women, from 9.1% to 18.3%. From 1979, it was possible to obtain data for the regions and it was observed that the lowest proportion was among men from the Northeast region in 1979 (5.4%) and the highest among women from the South region in 2017 (21.6%) (Figure 1).

Mortality rates for the age-adjusted set of cancers showed different magnitude patterns between regions of the country for both sexes or between men and women separately (Figure 1) and the trend was not constant. Oscillations were detected in this period of 38 years. Among men (1999–2017) there was a slight increase (APC = 0.14; p < 0.001); among women (2004–2017) the increase was greater (APC = 0.44; p < 0.001) (Table 1).

**Table 1:** Annual percentage change of standardized mortality rates for all cancer types among men and women residing in the Capitals and in the interior of Brazil and macro-regions, 1980 to 2017.

Geographical area	Both sexes			Men			Women		
	Period	APC	p	Period	APC	p	Period	APC	p
Brazil	1980–1990	-0.247	< 0.001	1980–1990	-0.136	0.007	1980–1989	-0.354	< 0.001
	1990–1997	0.504	< 0.001	1990–1999	0.415	< 0.001	1989–1996	0.530	< 0.001
	1997–2003	-0.175	0.001	1999–2017	0.142	< 0.001	1996–2004	-0.217	< 0.001
	2003–2017	0.241	< 0.001	–	–	–	2004–2017	0.443	< 0.001
Brazil, Capitals	1980–1990	-0.628	< 0.001	1980–1990	-0.581	< 0.001	1980–1989	-0.783	< 0.001
	1990–1997	0.520	< 0.001	1990–1997	0.619	< 0.001	1989–1996	0.624	< 0.001
	1997–2005	-1.535	< 0.001	1997–2005	-1.511	< 0.001	1996–2004	-1.487	< 0.001
	2005–2017	-0.460	< 0.001	2005–2017	-0.671	< 0.001	2004–2017	-0.241	< 0.001
Brazil, interior*	1980–1990	-0.051	0.195	1980–1990	0.026	0.643	1980–1990	-0.072	0.195
	1990–2017	0.536	< 0.001	1990–2017	0.582	< 0.001	1990–2017	0.584	< 0.001
North	1980–1991	-0.499	0.002	1980–1991	-1.027	< 0.001	1980–1991	-0.053	0.747
	1991–2009	0.905	< 0.001	1991–2009	1.020	< 0.001	1991–2009	0.812	< 0.001
	2009–2017	3.050	< 0.001	2009–2017	3.547	< 0.001	2009–2017	2.676	< 0.001
North, Capitals	1980–1991	-0.713	0.003	1980–1991	-1.073	< 0.001	1980–1991	-0.491	0.034
	1991–1999	0.688	0.017	1991–2000	0.512	0.109	1991–1998	0.996	0.002
	1999–2008	-1.495	< 0.001	2000–2009	-1.510	< 0.001	1998–2008	-1.370	< 0.001
	2008–2017	1.493	< 0.001	2009–2017	2.225	< 0.001	2008–2017	1.461	< 0.001
North, interior*	1980–1991	2.035	< 0.001	1980–1991	1.139	< 0.001	1980–1991	3.099	< 0.001
	1991–1999	0.565	0.052	1991–2000	0.986	0.001	1991–1998	0.019	0.963
	1999–2017	3.636	< 0.001	2000–2017	3.897	< 0.001	1998–2017	3.442	< 0.001
Northeast	1980–1993	0.935	0.086	1980–1996	1.264	0.053	1980–1993	0.231	0.062
	1993–2017	2.323	< 0.001	1996–2017	2.989	< 0.001	1993–2017	2.091	< 0.001
Northeast, capitals	1980–1986	-0.566	0.038	1980–1995	0.546	< 0.001	1980–1986	-1.580	< 0.001
	1986–1995	0.425	0.002	1995–2009	-0.434	< 0.001	1986–1993	0.667	0.001
	1995–2009	-0.729	< 0.001	2009–2017	1.894	< 0.001	1993–2008	-0.933	< 0.001
	2009–2017	1.455	< 0.001	–	–	–	2008–2017	1.200	< 0.001
Northeast, interior*	1980–1983	7.238	< 0.001	1980–1995	1.350	0.149	1980–1993	1.159	0.182
	1983–1995	0.044	0.959	1995–2017	3.796	< 0.001	1993–2017	3.050	< 0.001
	1995–2017	3.346	< 0.001	–	–	–	–	–	–
Southeast	1980–1989	-0.809	< 0.001	1980–1990	-0.532	< 0.001	1980–1988	-0.844	< 0.001
	1989–1996	0.418	0.047	1990–1997	0.473	< 0.001	1988–1996	0.401	< 0.001
	1996–2017	-0.664	< 0.001	1997–2017	-0.789	< 0.001	1996–2005	-1.046	< 0.001
	–	–	–	–	–	–	2005–2017	-0.054	0.248
Southeast, Capitals	1980–1989	-0.943	< 0.001	1980–1990	-0.614	< 0.001	1980–1988	-0.937	< 0.001
	1989–1996	0.615	0.030	1990–1997	0.578	< 0.001	1988–1996	0.568	< 0.001
	1996–2017	-1.213	< 0.001	1997–2017	-1.521	< 0.001	1996–2005	-1.380	< 0.001
	–	–	–	–	–	–	2005–2017	-0.502	< 0.001
Southeast, interior*	1980–1989	-0.577	< 0.001	1980–1990	-0.441	< 0.001	1980–1987	-0.877	< 0.001
	1989–1996	0.304	0.001	1990–1997	0.543	< 0.001	1987–1996	0.326	< 0.001
	1996–2017	-0.394	< 0.001	1997–2017	-0.471	< 0.001	1996–2007	-0.693	< 0.001
	–	–	–	–	–	–	2007–2017	0.307	< 0.001
South	1980–1984	-1.570	< 0.001	1980–1984	-1.028	< 0.001	1980–1984	-1.985	< 0.001
	1984–1993	0.600	< 0.001	1984–1993	0.544	< 0.001	1984–1994	0.707	< 0.001
	1993–2017	-0.555	< 0.001	1993–2010	-0.416	< 0.001	1994–2002	-0.784	< 0.001
	–	–	–	2010–2017	-1.466	< 0.001	2002–2017	-0.217	0.003
South, Capitals	1980–1998	-0.234	0.007	1980–1998	-0.294	0.005	1980–1984	-1.956	0.014
	1998–2017	-1.305	< 0.001	1998–2017	-1.460	< 0.001	1984–1997	0.221	0.187
	–	–	–	–	–	–	1997–2017	-1.178	< 0.001
South, interior*	1980–1984	-1.490	< 0.001	1980–1984	-0.973	< 0.001	1980–1984	-1.885	< 0.001
	1984–1993	0.643	< 0.001	1984–1993	0.637	< 0.001	1984–1994	0.707	< 0.001
	1993–2017	-0.454	< 0.001	1993–2010	-0.329	< 0.001	1994–2006	-0.558	< 0.001
	–	–	–	2010–2017	-1.339	< 0.001	2006–2017	0.095	0.340
Midwest	1980–1999	0.937	< 0.001	1980–1985	3.155	< 0.001	1980–1999	0.548	< 0.001
	1999–2017	0.166	0.490	1985–2017	0.634	< 0.001	1999–2007	-0.374	0.233
		–	–	–	–	–	2007–2017	0.572	0.076
		–	–	–	–	–	–	–	–
Midwest, Capitals	1980–1991	-1.410	< 0.001	1980–1990	-1.649	< 0.001	1980–2017	-0.587	< 0.001
	1991–1995	2.371	< 0.001	1990–1995	2.727	< 0.001	–	–	–
	1995–2017	-0.699	< 0.001	1995–2017	-0.569	< 0.001	–	–	–
Midwest, Capitals	1980–1989	3.098	0.002	1980–1985	6.358	< 0.001	1980–1999	1.421	0.001
	1989–2017	0.853	0.007	1985–2017	1.004	< 0.001	1999–2017	0.526	0.255

APC: annual percentage change in standardized mortality rates.

* Interior refers to municipalities outside the federal capitals.

Disaggregating by place of residence, four trends were seen for both sexes in the Capitals (declining until 1990, increasing from 1990 to 1997, sharp fall from 1997 to 2005 and lower intensity fall from 2005 to 2017) and only two in the interior (stability until 1990 and increase until 2017). Among men, it was in the Midwest region that the greatest fall was observed, which occurred in the Capitals (1980-1991: APC = −1.65; p < 0.001), and the greatest increase, which occurred in the other municipalities (1980-1985: APC = 6.36; p < 0.001). Among women, the greatest decline occurred in the southern region, both in the Capitals and in the interior, between 1980 and 1984 (APC = −1.96; p < 0.014 and APC = −1.89; p < 0.001, respectively), while the greatest growth occurred among residents of the interior of the northern region (1999-2017: APC = 3.44; p < 0.001).

Among men, lung, prostate, colorectal and stomach cancers accounted for 44.9% of all cancer deaths in 1979 and 43.4% in 2017. Among women, breast, lung, colorectal and cervical cancers accounted for 39.0% of cancer deaths in 1979 and 44.4% in 2017. Figure 3 shows the specific trends for these types of cancer and Table 2 shows the respective APC in the period studied.

**Figure 2:**
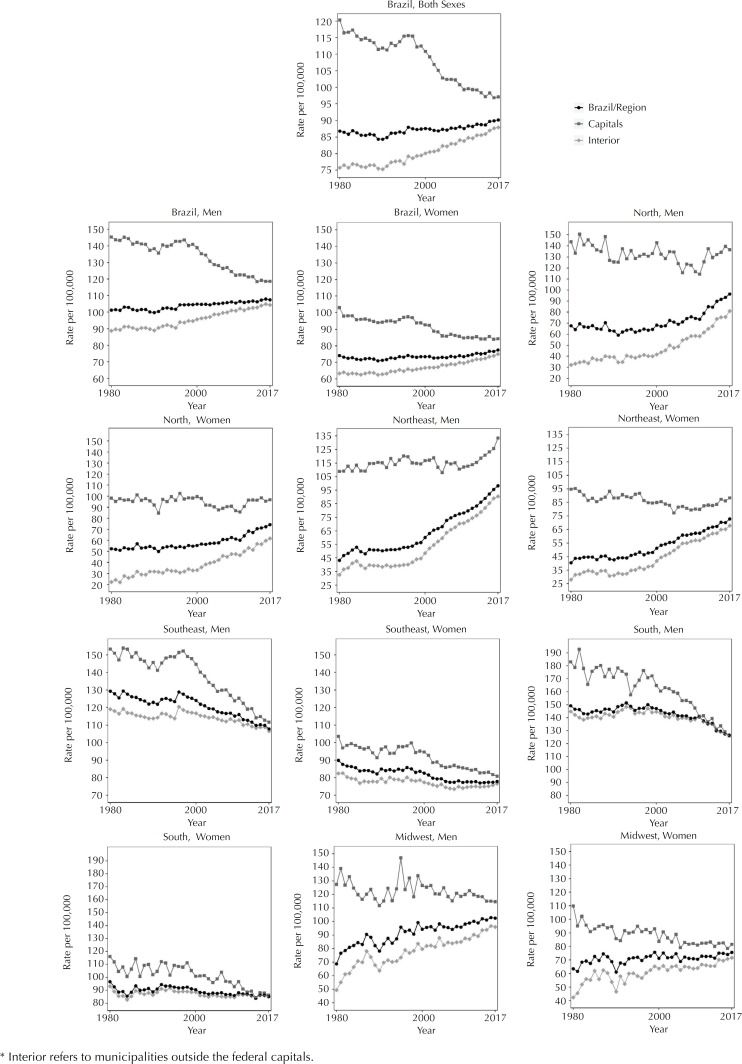
Temporal trend of mortality due to the set of cancers among men and women residing in the Capitals and in the interior* of Brazil and macro-regions, 1980 to 2017.

**Figure 3:**
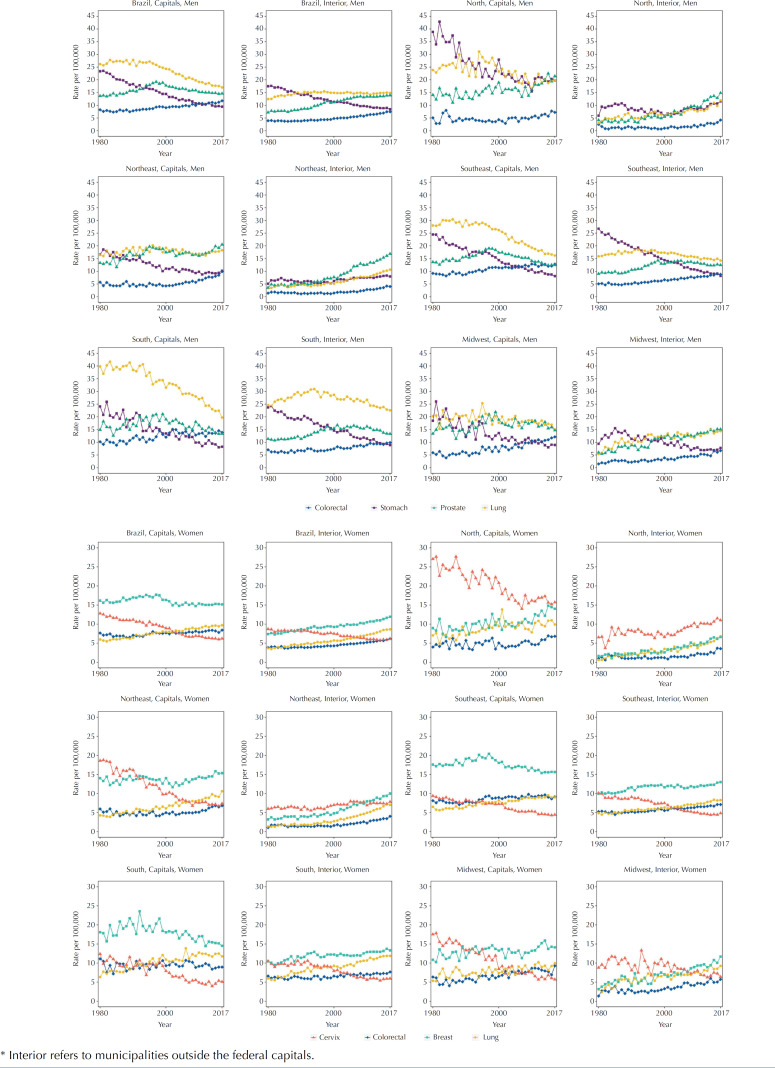
Temporal trend of mortality by specific types of cancer among men and women residing in the Capitals and in the interior* of Brazil and macro-regions, 1980 to 2017.

**Table 2:** Annual percentage change of standardized mortality rates for the most frequent types of cancer among men and women residing in the Capitals and in the interior of Brazil and macro-regions, 1980 to 2017.

Sex	Area	Lung cancer			Prostate cancer			Colorectal cancer			Stomach cancer		
		Period	APC	p	Period	APC	p	Period	APC	p	Period	APC	p
Men	Brazil	1980–1994	0.888	< 0.001	1980–1993	1.55	< 0.001	1980–1990	-0.205	0.156	1980–2017	-2.139	< 0.001
		1994–2011	-0.889	< 0.001	1993–1998	4.473	< 0.001	1990–2017	2.111	< 0.001	–	–	–
		2011–2017	-0.027	0.894	1998–2017	0.436	< 0.001	–	–	–	–	–	–
								–	–	–	–	–	–
	North. Capitals	1980–1996	0.772	0.040	1980–1992	-0.332	0.446	1980–1984	10.390	0.029	1980–2010	-2.473	< 0.001
		1996–2010	-2.664	< 0.001	1992–1999	3.742	< 0.001	1984–1999	-2.645	0.001	2010–2017	2.503	0.018
		2010–2017	1.048	0.329	1999–2007	-2.046	0.001	1999–2017	3.864	< 0.001	–	–	–
		–	–	–	2007–2017	4.003	< 0.001	–	–	–	–	–	–
	North. interior*	1980–2011	2.176	< 0.001	1980-2004	2.994	< 0.001	1980-1999	-2.213	0.009	1980–1985	7.593	< 0.001
		2011–2017	5.251	0.001	2004-2017	6.706	< 0.001	1999-2017	7.825	< 0.001	1985–2001	-3.071	< 0.001
		–	–	–	–	–	–	–	–	–	2001–2017	3.677	< 0.001
	Northeast. capitals	1980–1997	0.986	< 0.001	1980–1996	2.629	< 0.001	1980–2000	-0.715	0.008	1980–1999	-2.517	< 0.001
		1997–2010	-1.153	< 0.001	1996–2010	-1.191	< 0.001	2000–2017	4.342	< 0.001	1999–2017	-1.260	< 0.001
		2010–2017	1.258	0.001	2010–2017	3.420	< 0.001	–	–	–	–	–	–
	Northeast. interior*	1980–1994	1.436	0.036	1980-1993	2.067	< 0.001	1980-2000	-0.908	0.006	1980–1983	7.546	< 0.001
		1994–2017	3.637	< 0.001	1993-2017	5.095	< 0.001	2000-2017	6.964	< 0.001	1983–1996	-1.488	< 0.001
		–	–	–	–	–	–	–	–	–	1996–2017	2.082	< 0.001
	Southeast. Capitals	1980–1984	1.814	0.001	1980–1997	2.261	< 0.001	1980–1989	-0.038	0.913	1980–2017	-2.875	< 0.001
		1984–1996	-0.374	0.003	1997–2017	-2.056	< 0.001	1989–1997	2.897	< 0.001	–	–	–
		1996–2017	-2.714	< 0.001	–	–	–	1997–2017	0.628	< 0.001	–	–	–
	Southeast. interior*	1980–1994	0.981	< 0.001	1980–1988	0.199	0.485	1980–1987	-0.674	0.004	1980–2017	-3.048	< 0.001
		1994–2017	-1.102	< 0.001	1988–1997	3.870	< 0.001	1987–2017	2.075	< 0.001	–	–	–
		–	–	–	1997–2004	0.575	0.029	–	–	–	–	–	–
		–	–	–	2004–2017	-0.933	< 0.001	–	–	–	–	–	–
	South. Capitals	1980–1992	0.111	0.652	1980–1997	1.800	< 0.001	1980–1995	1.085	0.003	1980–2017	-2.766	< 0.001
		1992–2017	-2.318	< 0.001	1997–2017	-1.848	< 0.001	1995–2002	3.107	< 0.001	–	–	–
		–	–	–	–	–	–	2002–2017	-0.344	0.337	–	–	–
	South. interior*	1980–1993	1.669	< 0.001	1980–1990	0.733	0.002	1980–1985	-1.965	0.017	1980–2017	-2.594	< 0.001
		1993–2017	-1.173	< 0.001	1990–1998	3.590	< 0.001	1985–2017	1.525	< 0.001	–	–	–
		–	–	–	1998–2007	0.481	0.033	–	–	–	–	–	–
		–	–	–	2007–2017	-1.851	< 0.001	–	–	–	–	–	–
	Midwest. Capitals	1980–1995	0.507	0.064	1980–1990	-0.995	0.192	1980–1984	-5.501	0.014	1980–2017	-2.395	< 0.001
		1995–2017	-0.996	< 0.001	1990–1997	4.911	< 0.001	1984–2017	2.823	< 0.001	–	–	–
		–	–	–	1997–2017	-1.098	< 0.001	–	–	–	–	–	–
	Midwest. interior*	1980–1987	8.511	< 0.001	1980–1999	3.591	< 0.001	1980–1985	12.669	< 0.001	1980–1986	4.715	< 0.001
		1987–1996	1.614	0.006	1999–2017	1.620	< 0.001	1985–1992	-2.055	0.025	1986–2017	-2.426	< 0.001
		1996–2017	0.785	0.002	–	–	–	1992–2017	3.845	< 0.001	–	–	–
Women	Brazil	1980–1995	1.192	< 0.001	1980–2017	2.047	< 0.001	1980–1989	-0.831	< 0.001	1980–2017	-1.353	< 0.001
		1995–2004	-0.368	< 0.001	–	–	–	1989–2017	1.293	< 0.001	–	–	–
		2004–2017	1.016	< 0.001	–	–	–	–	–	–	–	–	–
	North. Capitals	1980–2005	0.731	0.010	1980–2001	2.600	< 0.001	1980–2003	-0.217	0.620	1980–2017	-1.596	< 0.001
		2005–2017	2.898	< 0.001	2001–2009	-1.738	0.066	2003–2017	2.753	< 0.001	–	–	–
		–	–	–	2009–2017	2.284	0.076	–	–	–	–	–	–
	North. interior*	1980–1998	2.876	< 0.001	1980–2017	5.002	< 0.001	1980–2000	-0.798	0.244	1980–1986	6.768	< 0.001
		1998–2017	4.855	< 0.001	–	–	–	2000–2017	6.428	< 0.001	1986–2002	-0.526	0.282
		–	–	–	–	–	–	–	–	–	2002–2017	3.209	< 0.001
	Northeast. capitals	1980–1984	-2.215	0.001	1980–2017	2.431	< 0.001	1980–1987	-3.286	< 0.001	1980–2007	-3.366	< 0.001
		1984–1993	1.434	< 0.001	–	–	–	1987–2008	0.359	0.021	2007–2017	-1.367	0.002
		1993–2003	-1.610	< 0.001	–	–	–	2008–2017	4.234	< 0.001	–	–	–
		2003–2017	1.715	< 0.001	–	–	–	–	–	–	–	–	–
	Northeast. interior*	1980–1999	2.005	< 0.001	1980–1993	2.438	< 0.001	1980–1999	-0.250	0.446	1980–1994	-0.469	0.026
		1999–2017	4.404	< 0.001	1993–2017	5.701	< 0.001	1999–2017	5.492	< 0.001	1994–2006	2.218	< 0.001
		–	–	–	–	–	–	–	–	–	2006–2017	-0.432	0.133
	Southeast. Capitals	1980–1996	0.743	< 0.001	1980–2007	1.491	< 0.001	1980–1989	-1.107	0.001	1980–2017	-2.051	< 0.001
		1996–2017	-1.189	< 0.001	2007–2017	0.733	0.020	1989–1997	2.578	< 0.001	–	–	–
		–	–	–	–	–	–	1997–2017	0.152	0.192	–	–	–
	Southeast. interior*	1980–1996	1.437	< 0.001	1980–2017	1.538	< 0.001	1980–1987	-0.955	0.004	1980–2017	-2.072	< 0.001
		1996–2007	-0.489	< 0.001	–	–	–	1987–2017	1.214	< 0.001	–	–	–
		2007–2017	0.986	< 0.001	–	–	–	–	–	–	–	–	–
	South. Capitals	1980–1990	2.031	< 0.001	1980–1997	2.621	< 0.001	1980–1983	-6.830	0.010	1980–1997	-1.449	0.001
		1990–2017	-1.223	< 0.001	1997–2017	0.780	< 0.001	1983–2007	0.621	0.004	1997–2017	-3.703	< 0.001
		–	–	–	–	–	–	2007–2017	-1.487	0.015	–	–	–
	South. interior*	1980–1993	1.786	< 0.001	1980–1994	3.166	< 0.001	1980–1985	-1.971	0.004	1980–1991	0.034	0.961
		1993–2006	-0.179	0.253	1994–2003	0.553	0.048	1985–2017	0.737	< 0.001	1991–2017	-2.306	< 0.001
		2006–2017	1.149	< 0.001	2003–2017	2.092	< 0.001	–	–	–	–	–	–
	Midwest. Capitals	1980–1997	1.351	< 0.001	1980–1992	1.321	0.046	1980–1985	-3.553	0.028	1980–2017	-3.158	< 0.001
		1997–2007	-1.262	0.007	1992–2017	0.909	0.001	1985–2009	2.212	< 0.001	–	–	–
		2007–2017	2.049	< 0.001	–	–	–	2009–2017	-0.096	0.916	–	–	–
	Midwest. Capitals	1980–2017	2.578	< 0.001	1980–1995	4.544	< 0.001	1980–1984	17.581	< 0.001	1980–1985	4.360	0.038
		–	–	–	1995–2017	1.276	< 0.001	1984–1991	-5.185	< 0.001	1985–2017	-1.524	< 0.001
		–	–	–	–	–	–	1991–2017	3.631	< 0.001	–	–	–

APC: annual percentage change in standardized mortality rates.

* Interior refers to municipalities outside the federal capitals.

The cancers that showed the greatest decline in mortality were stomach and cervix. Markedly, stomach cancer in men declined in all regions throughout the period, with the exception of the northern region, where a tendency to increase was seen in the Capitals after 2010 and in the interior from 2001, and in the interior of the Northeast region between 1996 and 2017, as shown by the APC in the period.

Among women, stomach cancer represented the seventh most frequent type of cancer in 2017 and, although the rates were much lower than those of men, the same decline occurred throughout the period, with the exception of the interior of the northern region, where there was an increase in the trend from 1980 to 1987, a fall from 1987 to 2001 and a resumption of growth from 2001 to 2017. Among residents of the interior of the Northeast region, there was a fall from 1980 to 1991 and an increase from 1991 to 2007, followed by stability thereafter (data not presented).

Cervical cancer, which was the second most frequent among female deaths in 1980, rose to fourth place at the end of the period. Although rates in the Capitals were higher, they were descending in all regions. It is noteworthy that, in general, there was no difference in the magnitude and trend of rates of this type of cancer among residents of the Capitals or the interior of the Southeast and South regions. The APC analysis indicated a fall both in the Capitals and in the interior of all regions, except in the interior of the North region between 2002 and 2017 and in the interior of the Northeast region between 1994 and 2006. In the interior of the Northeast region, however, as of since 2006 rates became stable, which did not happen for residents outside the Capitals in the North region.

Lung cancer had a different behavior according to sex and between the macro-regions and within them. Among men in the Capitals, rates began to fall from 1984 in the Southeast region, 1992 in the South Region, and 1995 in the Midwest region. Within the South-East and South regions, rates declined from 1994 and 1993, respectively. In the capitals of the North and Northeast regions, there was an unsustainable fall and a marked increase in the interior, especially from 2011 to 2017 in the North, and from 1994 to 2017 in the Northeast. In the Midwest region, the rates decreased in the Capitals between 1995 and 2017 and increased in the interior, but with intensity tending to decrease. Among women, there was a generalized tendency to increased mortality from lung cancer throughout the period, with the highest rates observed in the Southern region and the greatest increase in the temporal trend within the northern region throughout the series and within the Northeast region between 1993 and 2017.

Breast cancer in the southern region showed an increase in the Capitals until 1990, and after that a fall until 2017, which did not occur in the interior. In the Capitals of the Southeast region, there was a decline from 1996, while in the interior the fall that occurred between 1996 and 2007 did not continue afterwards. In the North and Northeast regions there was a tendency to increase, especially high in the interior.

Prostate cancer rates have always been higher in the Capitals than in the interior, especially in the North, Northeast and Midwest regions. The largest increases were observed in the interior of the northern regions between 2004 and 2017 and Northeast between 1993 and 2017. In the interior of the Midwest region, there was also a growing trend throughout the period. In the Capitals, between 1997 and 2017, rates declined in the Southeast, South and Midwest regions. Later, declining rates were observed in the interior of the Southeast region between 2004 and 2017, and the South region between 2007 and 2017.

Colorectal cancer was ranked sixth among men and fourth among women throughout the country at the beginning of the period and began to occupy the fourth position among men and third among women at the end. The highest rates were observed in the Southeast and South regions, both for men and women. Among men, from 1999, it was possible to notice an increasing trend in the North region, and from 2000 in the Northeast region, both in capitals and in the interior. In the Midwest region, there was an increase in the Capitals between 1984 and 2017 and in the interior between 1992 and 2017. An increasing trend was also observed in the Southeast and South regions, with the exception of residents of the capitals of the southern region, which from 2002 to 2017 showed rate stability.

Among women, there was also a tendency to increased mortality from colorectal cancer, both in the Capitals and in the interior of all regions, with the exception of the Capitals of the Southeast region, where rates remained stable between 1997 and 2017, and of the Midwest region, with stability between 2009 and 2017. Only in the capitals of the southern region there was a fall from 2007 to 2017.

It is noteworthy that the largest increases in the last two decades have been seen in the interior of the North and Northeast regions, both in men and women. In the interior of the northern region, it occurred for men between 1999 and 2017 (APC = 7.83; p < 0.001) and in the Northeast region, between 2000 and 2017 (APC = 6.96; p < 0.001). For women, the increase in the interior of the northern region occurred between 2000 and 2017 (APC = 6.42; p < 0.001) and in the interior of the Northeast region, between 1999 and 2017 (APC = 5.49; p < 0.001).

## DISCUSSION

Cancer mortality in Brazil for both sexes at the end of the study period (90.2/100,000 inhabitants) was similar to that of high-income countries (USA: 91.0/100,000, Canada: 92.8/100,000, United Kingdom: 102.6/100,000, Japan: 85.2/100,000)^16^. The most frequent types of cancer among deaths in the country, with the exception of cervical cancer, are also the most frequent in these countries (lung, breast, prostate, colorectal)^4^. The temporal trend of almost 40 years observed in the country as a whole for all cancers was not constant, with a fall in men between 1980 and 1990, followed by an increase between 1990 and 1999 that continued less pronounced until 2017. Among women there was a slight fall between 1980 and 1989, followed by an increase until 1996 and a fall until 2004, when rates rose again.

Data from other countries, mostly developed, point to the marked decrease in cancer mortality achieved in the last two decades^3^^,^^17^, attributed to preventive interventions, introduction of screening and, especially, to advances in diagnostic and therapeutic procedures^18^. However, advances in medicine are not always accessible to all individuals, especially in contexts of socioeconomic inequalities^19^.

The greatest fall detected in the study period occurred for stomach cancer in all regions, with the exception of the orthern region as a whole and the interior of the Northeast region. The decline, already reported in previous studies^5^, can be explained by a lower exposure to known carrcinogenic factors such as salty food intake and better food preservation^20^. However, there is a need to better understand the increase observed among residents of the northern Region and municipalities outside the Capitals in the Northeast regions.

Cervical cancer declined in all regions, but not among women in the interior of the northern region, where rates in 2017 are three times more frequent than in the Southeast region. Some effect of screening based on Pap smear, introduced throughout the country from 1992^21^, was able to reverse, in part, the trend of rate growth, but there are still women without access to screening in the interior of the North and Northeast regions, a fact reported previously with data until 2011^11^.

The inverse correlation between the Human Development Index (HDI) and national estimates of cervical cancer is well reported in the literature^4^. The U.S. cervical cancer mortality ratio among poorer and wealthier localities reached 2 between 2012 and 2016^22^. Several indicators can exemplify the inequality in mortality from this type of cancer among Brazilian regions, for example, the number of potential years of healthy life lost at 60 years of age is twice as high in the North region compared to the Southeast region^23^.

The South region has the highest municipal Human Development Index (HDI) in the country (0.766), while the North and Northeast regions, despite having had more accelerated growth between 2000 and 2010, have the lowest^24^. Although there was an improvement in health indicators in Brazil between 1990 and 2016, the burden of disease remains higher in the states of the North and Northeast regions compared to the Southeast and South^25^.

The experience of high-income countries, such as those in North America, part of Europe and Japan, is an example that investment in effective screening programs has made cervical cancer a rare disease^26^. Secondary prevention with the use of oncotic cytology, however, presupposes the structured operation of a care network with laboratory quality. Added to this, the effect of the human papillomavirus (HPV) vaccine will have long-term effects and does not replace screening for the disease. In this sense, the introduction of the HPV detection test can accelerate the effectiveness of screening, ^27^ and should be an option to consider in countries such as Brazil, which still has high rates of the disease.

Demographic changes in Brazil, with progressive population aging, increased life expectancy and reduced fertility, added to the increase in obesity^28^, have as a consequence an increase in the incidence of breast cancer. Although the incidence of breast cancer in Brazil is lower than in the USA and other European countries, mortality from this cancer is higher in all age groups, as a consequence of the high prevalence of cases diagnosed in advanced stage^29^. However, even with a tendency to increase the incidence, mortality rates declined in the capitals of the southern region from 1990 to 2017, and Southeast from 1996 to 2017, which may be an effect of better access to diagnosis and treatment of women living in these capitals^11^.

This same trend of decrease in the more developed regions of Brazil happens in relation to prostate cancer, for which there is a decline among residents in the capitals since 1997 and later among those of the interior. This trend may be related to the possibilities of specialized services offer for the treatment of early diagnosed cases^30^.

The increase in the incidence of prostate cancer in developed countries seems to be related to the increase in access to health services, as well as to the increase in the registration of cases^31^. An independent increase in the adoption of the routine prostate-specific antigen (PSA) detection test was observed, suggesting the interference of the western lifestyle, which leads to increased obesity and physical inactivity^32^ Although it is unclear why mortality rates have been falling in most Western countries, this fall has been attributed to early detection and improvement in treatment. The introduction of PSA test may have influenced early detection and thus reduced mortality. However, overscreening, which can lead to undesirable effects of treatment, contradicts the use of this test as a screening program^31^. The fact that the highest APC of prostate cancer mortality rates have been observed in the North and Northeast regions may suggest that there is a rapid absorption of the sedentary lifestyle coexisting with a low capacity to offer specialized services for diagnosis and treatment^30^.

Lung cancer was declining among men from the Southeast and South regions and in residents of the Capitals of the Midwest region. This, however, was not observed among women, for whom, in a generalized way, the rates were ascending in the period studied. In this case, because it is a cancer with still high lethality, the decrease in mortality occurs as a function of the decrease in incidence. The increased mortality from lung cancer among women has already been identified in studies in previous periods^33^^,^^34^.

The prevalence of smokers in Brazil decreased by 19% between 2008 and 2013, with a decline in all regions, in urban and rural areas, and in most states. There was a drop in all educational levels, but the highest prevalence was seen among blacks and mixed-race^35^. Smoking in Brazil is one of the lowest in the world and this may have a positive impact in the future, provided that control, regulation and prevention policies are maintained and strengthened^36^. Although the reduction in the prevalence of smokers occurred similarly in both sexes, it should be considered that the introduction of smoking among women occurred later; therefore, the increase in the occurrence of lung cancer still reflects this condition^36^.

Colorectal cancer, the third and fourth most frequent among women and men, respectively, shows an evolutionary pattern that deserves attention. Although rates in the Southeast and South regions are higher among men, they are three times higher than in the North.

The occurrence of colorectal cancer shows different patterns between countries. In several countries in Eastern Europe, Latin America and Asia, there was an upward trend in incidence and mortality. In Canada, European countries and Singapore, the incidence continues to increase, while there is a decline in mortality. In the group restricted to high HDI countries, there is a downward trend in both incidence and mortality^35^.

It is noteworthy that, in the North and Northeast regions, the trends in mortality from colorectal cancer increased more significantly in the interior, but there was no difference in magnitude between sexes. This may be an indication that the increase in obesity and sedentary lifestyle, classic risk factors for this type of tumors^37^, is occurring widely in the country. This increase may be a consequence of a real increase in the incidence, which, together with the lack of access to specialized diagnosis and treatment, may have an effect on mortality. Large differences in access to health services are seen between regions in Brazil, with a higher proportion of medical appointments in the South and Southeast, among people with better living conditions and in regions with higher HDI^30^.

The main limitation of this study stems from the quality of information on deaths in the country. Due to the great discrepancy in terms of completeness of information on the underlying cause of death, especially in the North and Northeast regions in the first two decades of the study period^12^^,^^13^, correction of ill-defined deaths and those registered as uterine cancer without other specification. Thus, all deaths registered as undefined underlying cause were distributed, which are classified in Chapter 16 of ICD – 9 from 1979 to 1995 and in the corresponding chapter of ICD-10 (18) from 1996.

Some previous studies^6^^,^^38^^,^^39^ follow the methodology proposed by the study Global Burden of Disease (GBD)^40^, which redistributes recorded deaths among causes considered as *garbage* codes. In this set of codes, in addition to the deaths recorded in chapters 16 of ICD-9 and 18 of ICD-10, other ill-defined causes and incomplete diagnoses from other chapters are included. In this study, we chose not to include the other nonspecific codes outside chapters 16 of ICD-9 and 18 of ICD – 10 due to the absence of studies that validate the contribution of redistribution of deaths recorded in the other nonspecific codes among specific types of cancer^41^. This option may have slightly underestimated the magnitude of the rates but did not detract from the comparison between regions in the period.

Previous studies that showed the trend of cancer in Brazil, with methodology applying correction of death data, included shorter periods than presented here: 1980-2006^5^, 1990-2015^6^ and 1996-2016^39^. In the case of cancer, it is important to understand the evolution of incidence and mortality for a longer period, as it was possible to show with data from SIM since 1978. To optimize the correction of death data, it was disaggregated according to place of residence, age group, sex and calendar year. For this reason, the trends presented here differ from those that do not correct the SIM data but are closer to detecting the directions and trend changes in cancer mortality. It should be emphasized that, in the two ICD classifications, the codes for the total number of malignant neoplasms and for the specific types selected, as well as the codes referring to the ill-defined root cause, are fully corresponding, which allowed to analyze the trend throughout the period with certainty that the use of the two versions did not introduce classification bias^42^.

Another aspect to be considered is the coverage of the death record. Although it has improved significantly in the country over these four decades, under-registration still exists in certain areas, especially in the states of the North and Northeast regions^43^^,^^44^. Since this study analyzed data from 1978 to 2017, it was decided not to perform the correction of the under-registration because no studies were found that evaluate the coverage of SIM from 2013 onwards. This option may have partially underestimated the rates calculated for the North and Northeast regions. However, since the main types of cancer occur from adulthood, and considering that most of the under-registration occurs in the age group up to one year of age^45^, the underestimation due to under-registration should not have been so high.

As for the application of linear regression by parts to evaluate the trend in time, it should be considered that, if the time interval is too small, trajectory changes may not present statistical significance due to the reduced number of points. However, this did not occur because, in the case of a long study period, the APC that were calculated covered periods of at least four years. In addition, the results of this study considered only those intervals that reached statistical significance. It should also be noticed that the number of deaths in each unit of analysis was not small, considering that the data were disaggregated to the level of the Capitals and the interior of each large region, which decreased the possibility of instability in the estimation of rates and of high heterogeneity in their distribution, that is, lower variance.

Another limitation of linear models for trend analysis is the possibility of residual autocorrelation. In this work, the models diagnosed with autocorrelation in residues were re-estimated via generalized least squares with a first-order autoregressive correlation structure-AR(1). No higher order correlation structures were identified. Therefore, when existing, autocorrelation was adequately treated in the model.

It is worth emphasizing that, at the same time as the reduction of some cancers related to poverty is observed, there is a real increase in new cases of cancer associated with factors arising from reproductive and hormonal history and diet^4^. Thus, for example, the time crossing point between cervical and breast cancer incidence curves is a typical indicator of cancer transition patterns in middle- and low-income countries^46^.

It was seen that, in the Southeast and South regions, this intersection between mortality from breast cancer and cervical cancer occurred before the 1980s among women living in the Capitals. Already in these same regions, for residents in the interior, this transition occurred later. Intermediate situations occurred in the Northeast and Midwest regions, where the crossing point of mortality between these two types of cancer in the Capitals occurred in 1992 and 1993, respectively. In the interior, however, it occurred only in 2008 in the Northeast region and in 2007 in the Midwest region. The most extreme situation is seen in the northern region, where this transition has not yet occurred either in the Capitals or in the interior, indicating a picture found in poor countries^4^.

In the same logic, among men, the crossing points of mortality curves for colorectal cancer, which is positively associated with HDI^35^, and stomach cancer, which is known to be related to poverty^4^, can be identified. It was in the capitals of the southern region that this crossing took place first; in 1998, the death rates from colorectal cancer exceeded those of stomach. In the same region, for those who live in the interior, this only happened in 2016. In the Southeast region this transition occurred in 2007 in the Capitals and only in 2016 in the interior. In the Midwest and Northeast regions, only in the Capitals the mortality rates from colorectal cancer in men exceeded those of stomach (2014 and 2017, respectively). The pattern of greater need was seen in the northern region, where stomach cancer rates are higher than those of colorectal cancer, both in the Capitals and in the interior.

Some results require a careful examination to understand the mechanisms involved both in relation to the accuracy of death information and in the quality of health care. The marked growth of prostate, colorectal and breast cancers within the North and Northeast regions deserves detailed investigation. One hypothesis would be an improvement in the diagnostic capacity, but with a high proportion of cases detected in late stages^29^. The hypothesis of improvement of the information on the underlying cause cannot also be ruled out, even considering that the correction was performed with redistribution of ill-classified deaths. It is possible that in these regions the correction was not enough to approach the actual weight of the occurrence of these tumors. Emphasis should also be given to the widespread growth of lung cancer in women, which was highly expressive in the interior of the North and Northeast regions.

Finally, the increase in rates for all cancers in recent years, particularly in the northern and northeastern regions, is worrying. This may mean a real increase in the incidence due to greater exposure to risk factors related to a sedentary lifestyle, but, in the case of mortality, the difficulties of access, especially to early detection and treatment of tumors of good prognosis such as breast and prostate, can lead to an increase in mortality.

Brazil is a country in economic transition and, during these four decades, has undergone several demographic, social and political modifications^47^, which interfere in the trend of specific types of cancer, configuring a specific transition pattern that assumes its own characteristics with important regional contrasts. The declines in mortality, birth and fertility levels observed since 1950 indicate that several changes have not occurred uniformly and simultaneously, but markedly shaped by socioeconomic inequality between and within regions^23^. In the Southeast, South and Central-West regions, this process was more accelerated, while in the North and Northeast, mortality and fertility levels were higher, with younger age structures ^47^.

Since the creation of the Unified Health System, incorporated into the Federal Constitution in 1988, there has been a great expansion of public services, with a significant improvement in health equity, but not enough to reduce extreme inequalities throughout the country^48^. In recent years, due to successive economic and political crises, some social indicators are already beginning to point to setbacks, and a scenario of uncertainty is configured for the coming years. A recent study, for example, concluded that the increase in unemployment in the country between 2012 and 2017 led to an excess of 30,000 deaths, mainly from cancer and cardiovascular diseases^49^. Demographic changes, socioeconomic inequalities and political crises that have followed in the country over four decades may partly explain the decrease in mortality in the Southeast and South regions and the increase among residents of municipalities outside the Capitals, especially in the North and Northeast regions.

The results found reflect the existence of distinct patterns of magnitude and trend of specific types of cancer that are sometimes contrasting and may reflect processes of illness and death with particular dynamics between the large regions of the country and between residents of capitals and other municipalities. While for residents of the capitals of the more developed regions there is a tendency to decreased mortality, in the poorest regions the significant increases, especially in the interior, make clear a profile of extreme inequity in health.
