# Supplementary material for: Cancer mortality in the Capitals and in the interior of Brazil: a four-decade analysis
Source: Rev Saude Publica. 2020 Nov 23;54:126. doi: 10.11606/s1518-8787.2020054002255 (PMC7688260; doi:10.11606/s1518-8787.2020054002255)
Supplement: Translation

# Translation: Mortalidade por câncer nas capitais e no interior do Brasil: uma análise de quatro décadas

**Authors:** Gulnar Azevedo e Silva, Beatriz Cordeiro Jardim, Vanessa de Melo Ferreira, Washington Leite Junger, Vania Reis Girianelli

**Affiliations:** I Rio de JaneiroRJ Brasil Universidade do Estado do Rio de Janeiro. Instituto de Medicina Social. Departamento de Epidemiologia. Rio de Janeiro, RJ, Brasil; II Rio de JaneiroRJ Brasil Instituto Nacional de Câncer José Alencar Gomes da Silva. Rio de Janeiro, RJ, Brasil; III Rio de JaneiroRJ Brasil Fundação Oswaldo Cruz. Escola Nacional de Saúde Pública Sérgio Arouca. Rio de Janeiro, RJ, Brasil

**Keywords:** Neoplasias, epidemiologia, Mortalidade, tendências, Estudos de Séries Temporais, Disparidades nos Níveis de Saúde

## Abstract

**OBJETIVO:**

Descrever a tendência da mortalidade por câncer geral e tipos mais frequentes entre homens e mulheres residentes nas capitais e demais municípios das cinco macrorregiões do Brasil entre 1978 e 2017.

**MÉTODOS:**

Estudo de séries temporais com dados de mortalidade corrigidos por redistribuição das causas maldefinidas. Foi calculada a mortalidade proporcional de câncer para Brasil e regiões. A variação percentual anual das taxas para o total de câncer e tipos específicos em cada segmento e na desagregação selecionada foi calculada por regressão linear generalizada com ligação gaussiana.

**RESULTADOS:**

A proporção de câncer aumentou progressivamente para ambos os sexos de 1978 a 2017. Diferenças importantes entre as capitais e o interior das macrorregiões foram vistas com dados desagregados. Os maiores declínios ocorreram para o câncer de estômago, exceto nas regiões Norte e interior da Nordeste, e de colo do útero, com queda generalizada, com exceção do interior da região Norte. O câncer de pulmão teve queda entre homens nas regiões Sudeste e Sul e aumento generalizado entre mulheres. Os cânceres de mama e de próstata tenderam a diminuir nas regiões Sudeste e Sul e entre residentes das capitais, mostrando, porém, aumento no interior das regiões Norte e Nordeste. O câncer colorretal teve tendência geral de aumento; nas capitais houve estabilidade entre homens da região Sul e entre mulheres das regiões Sudeste e Centro-Oeste e queda entre mulheres da região Sul a partir de 2007.

**CONCLUSÕES:**

A mortalidade por câncer apresentou grande variação entre os residentes de capitais e do interior das grandes regiões do país. Diminuição clara da mortalidade foi vista para os principais tipos nas regiões Sudeste e Sul. As regiões Norte e Nordeste apresentam padrões compatíveis com cânceres associados à pobreza ao mesmo tempo que se destacam pelo grande aumento daqueles relacionados ao estilo de vida sedentário.

## INTRODUÇÃO

O câncer é atualmente a segunda causa de adoecimento e morte no mundo, havendo grande variação na incidência e na mortalidade entre países em função do nível socioeconômico e da exposição a fatores de risco relativos a condições sociais e ao estilo de vida^1^. A ocorrência de câncer no mundo tem aumentado independentemente do crescimento demográfico; em 2012 foram estimados 14,1 milhões de casos novos e 8,2 milhões de óbitos, sendo esperado, para os próximos 20 anos, um crescimento mais expressivo na incidência mundial da doença^2^. Em países desenvolvidos, apesar de ainda haver aumento na incidência para alguns tipos de câncer, a mortalidade tem mostrado queda importante nas últimas duas décadas^3^.

A análise das tendências atuais de câncer indica que as transições econômicas e sociais em curso, em vários países de média e baixa renda, estão relacionadas ao aumento da exposição a fatores de risco reprodutivos e hormonais e a mudanças nos padrões alimentares^4^. O monitoramento da magnitude da incidência e da mortalidade por câncer em uma população é elemento fundamental para a definição de prioridades e ações direcionadas ao seu controle, bem como para a avaliação da eficácia das intervenções instituídas.

No Brasil, a mortalidade por câncer no início da década de 1980 ainda apresentava um padrão típico de países de média e baixa renda, com taxas expressivas de cânceres de colo do útero e estômago. Uma transição, entretanto, já se delineava, com aumento dos tipos associados às melhores condições socioeconômicas (mama, próstata e colorretal)^5^ marcado por padrões diferenciados entre as macrorregiões do país e entre moradores das capitais e demais municípios.

Embora tenham sido relatadas as tendências do câncer no Brasil e unidades federativas (UF) entre 1990 e 2015^6^, as diferenças existentes em relação ao local de residência, comparando-se os dados de habitantes das capitais com aqueles do interior nas cinco grandes regiões do país, ainda não foram exploradas em séries mais longas. Conhecer a evolução do câncer em função das mudanças que vêm ocorrendo nos padrões específicos de risco e nas possibilidades de acesso ao tratamento é fundamental para o aprimoramento de políticas regionalizadas de controle.

Além disso, deve ser considerado que o Sistema de Informação sobre Mortalidade (SIM) foi implementado no Brasil pelo Ministério da Saúde (MS) no final da década de 1970, tendo a declaração de óbito como documento-fonte. Desde então, o MS trabalha para aprimorar sua completitude e cobertura^7^. A série de dados consolidados disponível pelo SIM até 2017 permite a construção de séries históricas ampliadas, o que é aconselhável para o monitoramento dos diversos tipos de câncer.

Este estudo tem como objetivo descrever a tendência de quatro décadas (1978–2017) da mortalidade do conjunto de cânceres e dos tipos mais frequentes entre homens e mulheres residentes nas capitais e demais municípios das macrorregiões do Brasil.

## MÉTODOS

Trata-se de estudo de séries temporais que teve como fonte os dados do SIM referentes aos anos entre 1978^8^ e 2017^9^. Para o ano de 1978 foi utilizada a oitava codificação do Manual da Classificação Estatística Internacional de Doenças Lesões e Causas de Óbito (CID-8), entre 1979 e 1995 a nona (CID-9), e entre 1996 e 2017 a décima (CID-10).

Os denominadores populacionais foram os dos censos do Instituto Brasileiro de Geografia e Estatística (IBGE) para os anos de 1980, 1991, 2000 e 2010^9^. Para os anos intercensitários, foi feita interpolação linear. Para o período posterior (2011 a 2017), as projeções populacionais foram extrapoladas também linearmente, com desagregação por sexo, faixa etária e local de residência (UF e capitais) com base no período anterior.

Em homens os principais foram: pulmão (CID-9: 162; CID-10: C33-34), próstata (CID9: 185; CID10: C61), estômago (CID9: 151; CID10: C16), colorretal (CID-9: 153-154.1; CID-10: C18-20), e em mulheres foram: mama (CID-9: 170; CID-10: C50), pulmão (CID-9: 162; CID-10: C33-34), colorretal (CID-9: 153-154.1; CID-10: C18-20) e colo do útero (CID-9: 180; CID-10: C54). Foram excluídos os óbitos que tiveram informação ignorada para sexo, idade e município de residência. Como para o ano de 1978 foi calculada apenas a mortalidade proporcional por câncer, foram considerados os códigos CID referentes ao conjunto de cânceres (CID-8: 140-209) e o capítulo referente às causas maldefinidas (CID-8: cap. XVI).

A correção da informação sobre a causa básica de óbito foi feita seguindo metodologia proposta por Mathers et al.^10^ e a adaptação feita por Girianelli et al.^11^. Essa adaptação, que consiste em redistribuir proporcionalmente 50% dos óbitos com causa básica classificada como maldefinida (CID-8: cap. XVI, CID-9: 780-799; CID-10: R00-R99), é necessária porque o câncer é menos encontrado entre os óbitos maldefinidos do que entre os bem-definidos^12^. Dessa forma, os fatores de correção foram calculados para cada faixa etária (0–4, 5–14, 15–19, 20–29, 30–49, 50–59, 60–69, 70–79 e 80 anos ou mais), ano-calendário, sexo e local de residência (capitais e demais municípios) das cinco macrorregiões do país. Para os óbitos registrados como câncer do colo do útero, foi feita correção adicional com redistribuição de todos os óbitos classificados como neoplasia maligna do útero sem outra especificação (CID-9: 179; CID-10: C55), mantendo a proporção registrada como óbitos por câncer de colo e de corpo do útero^13^.

Com dados corrigidos, calculou-se a mortalidade proporcional por câncer para o Brasil. Como os dados de 1978 não contemplavam todas as regiões, a série de mortalidade proporcional para as macrorregiões foi iniciada em 1979.

Em seguida, foram calculadas as taxas de mortalidade por câncer brutas e padronizadas por idade (0–4, 5–14,15–19, 20–29, 30–49, 50–59, 60–69, 70–79 e 80 anos ou mais) e para cada sexo pelo método direto, tomando-se como população padrão a população mundial proposta por Segi^14^ para o período de 1980 a 2017. Essas taxas foram calculadas de forma desagregada por macrorregiões, capitais e demais municípios (interior).

Para estimar a tendência, foi utilizado modelo de regressão linear. Para séries com quebras estruturais, a variável tempo foi introduzida no modelo por meio de *spline* linear por partes, com pontos de quebra nos anos em que a tendência mudou sua trajetória. A identificação dos pontos de quebra foi visual e foram usadas *splines* cúbicas com 10 graus de liberdade para realçar os pontos de inflexão da curva, permitindo definir pontos de quebra em instantes específicos do tempo e a respectiva tendência em cada intervalo, ajustando-se por um segmento de reta contínuo entre os pontos. Optou-se por essa abordagem por se tratar de séries temporais com tendências que variam no tempo de forma não regular, o que torna inadequado um modelo linear para tendência global para todo o período.

Os modelos foram avaliados via resíduos para verificar se os pressupostos usuais foram atendidos, em particular a existência de autocorrelação residual por meio das funções de autocorrelação (FAC) e autocorrelação parcial (FACP). Os modelos que apresentaram autocorrelação nos resíduos significativa ou com valor absoluto maior que 0,5 foram reestimados usando mínimos quadrados generalizados com um modelo autorregressivo de primeira ordem AR(1) para modelar a autocorrelação e corrigir a variância dos estimadores dos coeficientes. Foi utilizada a função “gls” com estimação por máxima verossimilhança restrita (REML) do pacote “nlme”^15^.

O coeficiente do termo referente a cada segmento expressa o logaritmo da tendência naquele intervalo. Assim, foram calculadas as variações percentuais anuais (*annual percent change* – APC) das taxas de mortalidade pela fórmula e respectivos valores de p.

Para a interpretação das tendências, a não significância estatística (p-valor acima de 0,05) foi utilizada como critério para caracterizar uma APC como estável. A APC estatisticamente significante, quando positiva, indicou tendência crescente e, quando negativa, decrescente. As análises foram realizadas no Programa R, versão 3.4.1.

## RESULTADOS

A mortalidade proporcional por câncer aumentou de 1978 a 2017 em homens, de 8,3% para 16,2%, e em mulheres, de 9,1% para 18,3%. A partir de 1979, foi possível obter dados para as regiões e observou-se que a menor proporção foi entre homens da região Nordeste em 1979 (5,4%) e a maior entre mulheres da região Sul em 2017 (21,6%) (Figura 1).

**Figura 1:** Mortalidade proporcional por câncer, todos os tipos, em homens e mulheres no Brasil e macrorregiões, 1978 a 2017.

As taxas de mortalidade para o conjunto de cânceres ajustadas por idade mostraram padrões de magnitude diferentes entre as regiões do país para ambos os sexos ou entre homens e mulheres em separado (Figura 1) e a tendência não foi constante. Oscilações foram detectadas nesse período de 38 anos. Entre homens (1999–2017) houve leve aumento (APC = 0,14; p < 0,001); entre as mulheres (2004–2017) o aumento foi maior (APC = 0,44; p < 0,001) (Tabela 1).

**Tabela 1:** Variação percentual anual das taxas de mortalidade padronizadas para o conjunto de cânceres entre homens e mulheres residentes nas capitais e no interior do Brasil e macrorregiões, 1980 a 2017.

Área geográfica	Ambos os sexos			Homens			Mulheres		
	Período	APC	p	Período	APC	p	Período	APC	p
Brasil	1980–1990	-0,247	< 0,001	1980–1990	-0,136	0,007	1980–1989	-0,354	< 0,001
	1990–1997	0,504	< 0,001	1990–1999	0,415	< 0,001	1989–1996	0,530	< 0,001
	1997–2003	-0,175	0,001	1999–2017	0,142	< 0,001	1996–2004	-0,217	< 0,001
	2003–2017	0,241	< 0,001	–	–	–	2004–2017	0,443	< 0,001
Brasil, capitais	1980–1990	-0,628	< 0,001	1980–1990	-0,581	< 0,001	1980–1989	-0,783	< 0,001
	1990–1997	0,520	< 0,001	1990–1997	0,619	< 0,001	1989–1996	0,624	< 0,001
	1997–2005	-1,535	< 0,001	1997–2005	-1,511	< 0,001	1996–2004	-1,487	< 0,001
	2005–2017	-0,460	< 0,001	2005–2017	-0,671	< 0,001	2004–2017	-0,241	< 0,001
Brasil, interior*	1980–1990	-0,051	0,195	1980–1990	0,026	0,643	1980–1990	-0,072	0,195
	1990–2017	0,536	< 0,001	1990–2017	0,582	< 0,001	1990–2017	0,584	< 0,001
Norte	1980–1991	-0,499	0,002	1980–1991	-1,027	< 0,001	1980–1991	-0,053	0,747
	1991–2009	0,905	< 0,001	1991–2009	1,020	< 0,001	1991–2009	0,812	< 0,001
	2009–2017	3,050	< 0,001	2009–2017	3,547	< 0,001	2009–2017	2,676	< 0,001
Norte, capitais	1980–1991	-0,713	0,003	1980–1991	-1,073	< 0,001	1980–1991	-0,491	0,034
	1991–1999	0,688	0,017	1991–2000	0,512	0,109	1991–1998	0,996	0,002
	1999–2008	-1,495	< 0,001	2000–2009	-1,510	< 0,001	1998–2008	-1,370	< 0,001
	2008–2017	1,493	< 0,001	2009–2017	2,225	< 0,001	2008–2017	1,461	< 0,001
Norte, interior*	1980–1991	2,035	< 0,001	1980–1991	1,139	< 0,001	1980–1991	3,099	< 0,001
	1991–1999	0,565	0,052	1991–2000	0,986	0,001	1991–1998	0,019	0,963
	1999–2017	3,636	< 0,001	2000–2017	3,897	< 0,001	1998–2017	3,442	< 0,001
Nordeste	1980–1993	0,935	0,086	1980–1996	1,264	0,053	1980–1993	0,231	0,062
	1993–2017	2,323	< 0,001	1996–2017	2,989	< 0,001	1993–2017	2,091	< 0,001
Nordeste, capitais	1980–1986	-0,566	0,038	1980–1995	0,546	< 0,001	1980–1986	-1,580	< 0,001
	1986–1995	0,425	0,002	1995–2009	-0,434	< 0,001	1986–1993	0,667	0,001
	1995–2009	-0,729	< 0,001	2009–2017	1,894	< 0,001	1993–2008	-0,933	< 0,001
	2009–2017	1,455	< 0,001	–	–	–	2008–2017	1,200	< 0,001
Nordeste, interior*	1980–1983	7,238	< 0,001	1980–1995	1,350	0,149	1980–1993	1,159	0,182
	1983–1995	0,044	0,959	1995–2017	3,796	< 0,001	1993–2017	3,050	< 0,001
	1995–2017	3,346	< 0,001	–	–	–	–	–	–
Sudeste	1980–1989	-0,809	< 0,001	1980–1990	-0,532	< 0,001	1980–1988	-0,844	< 0,001
	1989–1996	0,418	0,047	1990–1997	0,473	< 0,001	1988–1996	0,401	< 0,001
	1996–2017	-0,664	< 0,001	1997–2017	-0,789	< 0,001	1996–2005	-1,046	< 0,001
	–	–	–	–	–	–	2005–2017	-0,054	0,248
Sudeste, capitais	1980–1989	-0,943	< 0,001	1980–1990	-0,614	< 0,001	1980–1988	-0,937	< 0,001
	1989–1996	0,615	0,030	1990–1997	0,578	< 0,001	1988–1996	0,568	< 0,001
	1996–2017	-1,213	< 0,001	1997–2017	-1,521	< 0,001	1996–2005	-1,380	< 0,001
	–	–	–	–	–	–	2005–2017	-0,502	< 0,001
Sudeste, interior*	1980–1989	-0,577	< 0,001	1980–1990	-0,441	< 0,001	1980–1987	-0,877	< 0,001
	1989–1996	0,304	0,001	1990–1997	0,543	< 0,001	1987–1996	0,326	< 0,001
	1996–2017	-0,394	< 0,001	1997–2017	-0,471	< 0,001	1996–2007	-0,693	< 0,001
	–	–	–	–	–	–	2007–2017	0,307	< 0,001
Sul	1980–1984	-1,570	< 0,001	1980–1984	-1,028	< 0,001	1980–1984	-1,985	< 0,001
	1984–1993	0,600	< 0,001	1984–1993	0,544	< 0,001	1984–1994	0,707	< 0,001
	1993–2017	-0,555	< 0,001	1993–2010	-0,416	< 0,001	1994–2002	-0,784	< 0,001
	–	–	–	2010–2017	-1,466	< 0,001	2002–2017	-0,217	0,003
Sul, capitais	1980–1998	-0,234	0,007	1980–1998	-0,294	0,005	1980–1984	-1,956	0,014
	1998–2017	-1,305	< 0,001	1998–2017	-1,460	< 0,001	1984–1997	0,221	0,187
	–	–	–	–	–	–	1997–2017	-1,178	< 0,001
Sul, interior*	1980–1984	-1,490	< 0,001	1980–1984	-0,973	< 0,001	1980–1984	-1,885	< 0,001
	1984–1993	0,643	< 0,001	1984–1993	0,637	< 0,001	1984–1994	0,707	< 0,001
	1993–2017	-0,454	< 0,001	1993–2010	-0,329	< 0,001	1994–2006	-0,558	< 0,001
	–	–	–	2010–2017	-1,339	< 0,001	2006–2017	0,095	0,340
Centro-Oeste	1980–1999	0,937	< 0,001	1980–1985	3,155	< 0,001	1980–1999	0,548	< 0,001
	1999–2017	0,166	0,490	1985–2017	0,634	< 0,001	1999–2007	-0,374	0,233
		–	–	–	–	–	2007–2017	0,572	0,076
		–	–	–	–	–	–	–	–
Centro-Oeste, capitais	1980–1991	-1,410	< 0,001	1980–1990	-1,649	< 0,001	1980–2017	-0,587	< 0,001
	1991–1995	2,371	< 0,001	1990–1995	2,727	< 0,001	–	–	–
	1995–2017	-0,699	< 0,001	1995–2017	-0,569	< 0,001	–	–	–
Centro-Oeste, interior*	1980–1989	3,098	0,002	1980–1985	6,358	< 0,001	1980–1999	1,421	0,001
	1989–2017	0,853	0,007	1985–2017	1,004	< 0,001	1999–2017	0,526	0,255

APC: variação percentual anual das taxas de mortalidade padronizadas.

* Interior refere-se aos municípios fora das capitais federativas.

Desagregando-se por local de residência, quatro tendências foram vistas para ambos os sexos nas capitais (declinante até 1990, aumento de 1990 a 1997, queda acentuada de 1997 a 2005 e queda de menor intensidade de 2005 a 2017) e apenas duas no interior (estabilidade até 1990 e aumento até 2017). Entre os homens, foi na região Centro-Oeste que se observou a maior queda, a qual aconteceu nas capitais (1980–1991: APC = −1,65; p < 0,001), e o maior aumento, que ocorreu nos demais municípios (1980–1985: APC = 6,36; p < 0,001). Entre mulheres, o maior declínio ocorreu na região Sul, tanto nas capitais quanto no interior, entre 1980 e 1984 (APC = −1,96; p < 0,014 e APC = −1,89; p < 0,001, respectivamente), enquanto o maior crescimento ocorreu entre as residentes do interior da região Norte (1999–2017: APC = 3,44; p < 0,001).

Entre homens, os cânceres de pulmão, próstata, colorretal e estômago corresponderam a 44,9% do total de óbitos por câncer em 1979 e 43,4% em 2017. Entre mulheres, os cânceres de mama, pulmão, colorretal e colo do útero totalizaram 39,0% dos óbitos por câncer em 1979 e 44,4% em 2017. Na Figura 3 são mostradas as tendências especificas para esses tipos de câncer e na Tabela 2, as respectivas APC no período estudado.

**Figura 2:** Tendência temporal da mortalidade pelo conjunto de cânceres entre homens e mulheres residentes nas capitais e no interior* do Brasil e macrorregiões, 1980 a 2017.

**Figura 3:** Tendência temporal da mortalidade por tipos específicos de câncer entre homens e mulheres residentes nas capitais e no interior* do Brasil e macrorregiões, 1980 a 2017.

**Tabela 2:** Variação percentual anual das taxas de mortalidade padronizadas para os tipos mais frequentes de câncer entre homens e mulheres residentes nas capitais e no interior do Brasil e macrorregiões, 1980 a 2017.

Sexo	Área	Câncer de pulmão			Câncer de próstata			Câncer colorretal			Câncer de estômago		
		Período	APC	p	Período	APC	p	Período	APC	p	Período	APC	p
Homens	Brasil	1980–1994	0,888	< 0,001	1980–1993	1,55	< 0,001	1980–1990	-0,205	0,156	1980–2017	-2,139	< 0,001
		1994–2011	-0,889	< 0,001	1993–1998	4,473	< 0,001	1990–2017	2,111	< 0,001	–	–	–
		2011–2017	-0,027	0,894	1998–2017	0,436	< 0,001	–	–	–	–	–	–
								–	–	–	–	–	–
	Norte, capitais	1980–1996	0,772	0,040	1980–1992	-0,332	0,446	1980–1984	10,390	0,029	1980–2010	-2,473	< 0,001
		1996–2010	-2,664	< 0,001	1992–1999	3,742	< 0,001	1984–1999	-2,645	0,001	2010–2017	2,503	0,018
		2010–2017	1,048	0,329	1999–2007	-2,046	0,001	1999–2017	3,864	< 0,001	–	–	–
		–	–	–	2007–2017	4,003	< 0,001	–	–	–	–	–	–
	Norte, interior*	1980–2011	2,176	< 0,001	1980-2004	2,994	< 0,001	1980-1999	-2,213	0,009	1980–1985	7,593	< 0,001
		2011–2017	5,251	0,001	2004-2017	6,706	< 0,001	1999-2017	7,825	< 0,001	1985–2001	-3,071	< 0,001
		–	–	–	–	–	–	–	–	–	2001–2017	3,677	< 0,001
	Nordeste, capitais	1980–1997	0,986	< 0,001	1980–1996	2,629	< 0,001	1980–2000	-0,715	0,008	1980–1999	-2,517	< 0,001
		1997–2010	-1,153	< 0,001	1996–2010	-1,191	< 0,001	2000–2017	4,342	< 0,001	1999–2017	-1,260	< 0,001
		2010–2017	1,258	0,001	2010–2017	3,420	< 0,001	–	–	–	–	–	–
	Nordeste, interior*	1980–1994	1,436	0,036	1980-1993	2,067	< 0,001	1980-2000	-0,908	0,006	1980–1983	7,546	< 0,001
		1994–2017	3,637	< 0,001	1993-2017	5,095	< 0,001	2000-2017	6,964	< 0,001	1983–1996	-1,488	< 0,001
		–	–	–	–	–	–	–	–	–	1996–2017	2,082	< 0,001
	Sudeste, capitais	1980–1984	1,814	0,001	1980–1997	2,261	< 0,001	1980–1989	-0,038	0,913	1980–2017	-2,875	< 0,001
		1984–1996	-0,374	0,003	1997–2017	-2,056	< 0,001	1989–1997	2,897	< 0,001	–	–	–
		1996–2017	-2,714	< 0,001	–	–	–	1997–2017	0,628	< 0,001	–	–	–
	Sudeste, interior*	1980–1994	0,981	< 0,001	1980–1988	0,199	0,485	1980–1987	-0,674	0,004	1980–2017	-3,048	< 0,001
		1994–2017	-1,102	< 0,001	1988–1997	3,870	< 0,001	1987–2017	2,075	< 0,001	–	–	–
		–	–	–	1997–2004	0,575	0,029	–	–	–	–	–	–
		–	–	–	2004–2017	-0,933	< 0,001	–	–	–	–	–	–
	Sul, capitais	1980–1992	0,111	0,652	1980–1997	1,800	< 0,001	1980–1995	1,085	0,003	1980–2017	-2,766	< 0,001
		1992–2017	-2,318	< 0,001	1997–2017	-1,848	< 0,001	1995–2002	3,107	< 0,001	–	–	–
		–	–	–	–	–	–	2002–2017	-0,344	0,337	–	–	–
	Sul, interior*	1980–1993	1,669	< 0,001	1980–1990	0,733	0,002	1980–1985	-1,965	0,017	1980–2017	-2,594	< 0,001
		1993–2017	-1,173	< 0,001	1990–1998	3,590	< 0,001	1985–2017	1,525	< 0,001	–	–	–
		–	–	–	1998–2007	0,481	0,033	–	–	–	–	–	–
		–	–	–	2007–2017	-1,851	< 0,001	–	–	–	–	–	–
	Centro-Oeste, capitais	1980–1995	0,507	0,064	1980–1990	-0,995	0,192	1980–1984	-5,501	0,014	1980–2017	-2,395	< 0,001
		1995–2017	-0,996	< 0,001	1990–1997	4,911	< 0,001	1984–2017	2,823	< 0,001	–	–	–
		–	–	–	1997–2017	-1,098	< 0,001	–	–	–	–	–	–
	Centro-Oeste, interior*	1980–1987	8,511	< 0,001	1980–1999	3,591	< 0,001	1980–1985	12,669	< 0,001	1980–1986	4,715	< 0,001
		1987–1996	1,614	0,006	1999–2017	1,620	< 0,001	1985–1992	-2,055	0,025	1986–2017	-2,426	< 0,001
		1996–2017	0,785	0,002	–	–	–	1992–2017	3,845	< 0,001	–	–	–
Mulheres	Brasil	1980–1995	1,192	< 0,001	1980–2017	2,047	< 0,001	1980–1989	-0,831	< 0,001	1980–2017	-1,353	< 0,001
		1995–2004	-0,368	< 0,001	–	–	–	1989–2017	1,293	< 0,001	–	–	–
		2004–2017	1,016	< 0,001	–	–	–	–	–	–	–	–	–
	Norte, capitais	1980–2005	0,731	0,010	1980–2001	2,600	< 0,001	1980–2003	-0,217	0,620	1980–2017	-1,596	< 0,001
		2005–2017	2,898	< 0,001	2001–2009	-1,738	0,066	2003–2017	2,753	< 0,001	–	–	–
		–	–	–	2009–2017	2,284	0,076	–	–	–	–	–	–
	Norte, interior*	1980–1998	2,876	< 0,001	1980–2017	5,002	< 0,001	1980–2000	-0,798	0,244	1980–1986	6,768	< 0,001
		1998–2017	4,855	< 0,001	–	–	–	2000–2017	6,428	< 0,001	1986–2002	-0,526	0,282
		–	–	–	–	–	–	–	–	–	2002–2017	3,209	< 0,001
	Nordeste, capitais	1980–1984	-2,215	0,001	1980–2017	2,431	< 0,001	1980–1987	-3,286	< 0,001	1980–2007	-3,366	< 0,001
		1984–1993	1,434	< 0,001	–	–	–	1987–2008	0,359	0,021	2007–2017	-1,367	0,002
		1993–2003	-1,610	< 0,001	–	–	–	2008–2017	4,234	< 0,001	–	–	–
		2003–2017	1,715	< 0,001	–	–	–	–	–	–	–	–	–
	Nordeste, interior*	1980–1999	2,005	< 0,001	1980–1993	2,438	< 0,001	1980–1999	-0,250	0,446	1980–1994	-0,469	0,026
		1999–2017	4,404	< 0,001	1993–2017	5,701	< 0,001	1999–2017	5,492	< 0,001	1994–2006	2,218	< 0,001
		–	–	–	–	–	–	–	–	–	2006–2017	-0,432	0,133
	Sudeste, capitais	1980–1996	0,743	< 0,001	1980–2007	1,491	< 0,001	1980–1989	-1,107	0,001	1980–2017	-2,051	< 0,001
		1996–2017	-1,189	< 0,001	2007–2017	0,733	0,020	1989–1997	2,578	< 0,001	–	–	–
		–	–	–	–	–	–	1997–2017	0,152	0,192	–	–	–
	Sudeste, interior*	1980–1996	1,437	< 0,001	1980–2017	1,538	< 0,001	1980–1987	-0,955	0,004	1980–2017	-2,072	< 0,001
		1996–2007	-0,489	< 0,001	–	–	–	1987–2017	1,214	< 0,001	–	–	–
		2007–2017	0,986	< 0,001	–	–	–	–	–	–	–	–	–
	Sul, capitais	1980–1990	2,031	< 0,001	1980–1997	2,621	< 0,001	1980–1983	-6,830	0,010	1980–1997	-1,449	0,001
		1990–2017	-1,223	< 0,001	1997–2017	0,780	< 0,001	1983–2007	0,621	0,004	1997–2017	-3,703	< 0,001
		–	–	–	–	–	–	2007–2017	-1,487	0,015	–	–	–
	Sul, interior*	1980–1993	1,786	< 0,001	1980–1994	3,166	< 0,001	1980–1985	-1,971	0,004	1980–1991	0,034	0,961
		1993–2006	-0,179	0,253	1994–2003	0,553	0,048	1985–2017	0,737	< 0,001	1991–2017	-2,306	< 0,001
		2006–2017	1,149	< 0,001	2003–2017	2,092	< 0,001	–	–	–	–	–	–
	Centro-Oeste, capitais	1980–1997	1,351	< 0,001	1980–1992	1,321	0,046	1980–1985	-3,553	0,028	1980–2017	-3,158	< 0,001
		1997–2007	-1,262	0,007	1992–2017	0,909	0,001	1985–2009	2,212	< 0,001	–	–	–
		2007–2017	2,049	< 0,001	–	–	–	2009–2017	-0,096	0,916	–	–	–
	Centro-Oeste, interior*	1980–2017	2,578	< 0,001	1980–1995	4,544	< 0,001	1980–1984	17,581	< 0,001	1980–1985	4,360	0,038
		–	–	–	1995–2017	1,276	< 0,001	1984–1991	-5,185	< 0,001	1985–2017	-1,524	< 0,001
		–	–	–	–	–	–	1991–2017	3,631	< 0,001	–	–	–

* Interior refere-se aos municípios fora das capitais federativas.

APC: variação percentual anual das taxas de mortalidade padronizadas

Os cânceres que apresentaram o maior declínio na mortalidade foram o de estômago e do colo do útero. Marcadamente, o câncer de estômago em homens declinou em todas as regiões por todo o período, com exceção da regiões Norte, onde tendência a aumento foi vista nas capitais após 2010 e no interior a partir de 2001, e do interior da região Nordeste entre 1996 e 2017, como mostram as APC no período.

Entre mulheres, o câncer de estomago representou o sétimo tipo de câncer mais frequente em 2017 e, embora, as taxas tenham sido bem inferiores do que as masculinas, o mesmo declínio se deu em todo o período, com exceção do interior da região Norte, onde houve aumento da tendência de 1980 a 1987, queda de 1987 a 2001 e retomada de crescimento de 2001 a 2017. Entre as residentes do interior da região Nordeste, houve queda de 1980 a 1991 e aumento de 1991 a 2007, seguido de estabilidade a partir daí (dados não apresentados).

O câncer do colo do útero, que se colocava como o segundo mais frequente entre os óbitos femininos em 1980, passou para o quarto lugar no final do período. Embora as taxas nas capitais tenham sido mais altas, elas foram descendentes em todas as regiões. Ressalta-se que, em geral, não houve diferença na magnitude e tendência das taxas desse tipo de câncer entre residentes das capitais ou do interior das regiões Sudeste e Sul. A análise das APC indicou queda tanto nas capitais quanto no interior de todas as regiões, exceto no interior da região Norte entre 2002 e 2017 e no interior da região Nordeste entre 1994 e 2006. No interior da região Nordeste, contudo, a partir de 2006 as taxas se tornaram estáveis, o que não aconteceu para as residentes fora das capitais na região Norte.

O câncer de pulmão teve um comportamento diferente segundo sexo e entre as macrorregiões e dentro delas. Entre os homens das capitais, as taxas começaram a cair a partir de 1984 na região Sudeste, 1992 na região Sul e 1995 na região Centro-Oeste. No interior das regiões Sudeste e Sul, as taxas declinaram a partir de 1994 e 1993, respectivamente. Nas capitais das regiões Norte e Nordeste, houve queda não sustentada e aumento marcante no interior, sobretudo de 2011 a 2017 no Norte e de 1994 a 2017 no Nordeste. Na região Centro-Oeste, houve queda nas capitais entre 1995 e 2017 e aumento das taxas no interior, mas com intensidade tendendo à diminuição. Já entre mulheres, houve tendência generalizada a aumento da mortalidade por câncer de pulmão em todo período, sendo as maiores taxas observadas na região Sul e o maior aumento na tendência temporal no interior da região Norte em toda a série e no interior da região Nordeste entre 1993 e 2017.

O câncer de mama na região Sul mostrou aumento nas capitais até 1990, e a partir daí houve queda até 2017, o que não se verificou no interior. Já nas capitais da região Sudeste, houve declínio a partir de 1996, ao passo que no interior a queda ocorrida entre 1996 e 2007 não se manteve depois. Nas regiões Norte e Nordeste houve tendência a aumento, especialmente elevado no interior.

As taxas do câncer de próstata foram sempre mais altas nas capitais que no interior, em especial nas regiões Norte, Nordeste e Centro-Oeste. Os maiores aumentos foram observados no interior das regiões Norte entre 2004 e 2017 e Nordeste entre 1993 e 2017. No interior da região Centro-Oeste, também foi verificada tendência crescente em todo o período. Já nas capitais, entre 1997 e 2017, as taxas declinaram nas regiões Sudeste, Sul e Centro-Oeste. Mais tardiamente, taxas em declínio foram verificadas no interior da região Sudeste entre 2004 e 2017 e da região Sul entre 2007 e 2017.

O câncer colorretal se colocava na sexta posição entre homens e quarta entre as mulheres em todo o país no início do período e passou a ocupar a quarta posição entre homens e terceira entre as mulheres ao final. As maiores taxas foram observadas nas regiões Sudeste e Sul, tanto para homens quanto para mulheres. Entre os homens, a partir de 1999, foi possível notar tendência de aumento na região Norte, e a partir de 2000 na região Nordeste, tanto em capitais quanto interior. Já na região Centro-Oeste, observou-se aumento nas capitais entre 1984 e 2017 e no interior entre 1992 e 2017. Tendência a aumento também foi verificada nas regiões Sudeste e Sul, com exceção dos moradores das capitais da região Sul, que de 2002 a 2017 mostraram estabilidade das taxas.

Entre as mulheres também houve tendência a aumento da mortalidade do câncer colorretal, tanto nas capitais quanto no interior de todas as regiões, com exceção das capitais da região Sudeste, onde as taxas mantiveram-se estáveis entre 1997 e 2017, e da região Centro-Oeste, com estabilidade entre 2009 e 2017. Apenas nas capitais da região Sul foi verificada queda de 2007 a 2017.

Chama atenção que os maiores aumentos nas duas últimas décadas foram verificados no interior das regiões Norte e Nordeste, tanto em homens quanto em mulheres. No interior da região Norte, ele se deu para os homens entre 1999 e 2017 (APC = 7,83; p < 0,001) e na região Nordeste, entre 2000 e 2017 (APC = 6,96; p < 0,001). Para as mulheres, o aumento no interior da região Norte aconteceu entre 2000 e 2017 (APC = 6,42; p < 0,001) e no interior da região Nordeste, entre 1999 e 2017 (APC = 5,49; p < 0,001).

## DISCUSSÃO

A mortalidade por câncer no Brasil para ambos os sexos no final do período estudado (90,2/100.000 habitantes) foi semelhante à de países de alta renda (EUA: 91,0/100.000, Canadá: 92,8/100.000, Reino Unido: 102,6/100.000, Japão: 85,2/100.000)^16^. Os tipos de câncer mais frequentes entre os óbitos no país, com exceção do câncer de colo do útero, são também os mais frequentes nesses países (pulmão, mama, próstata, colorretal)^4^. A tendência temporal de quase 40 anos observada no país como um todo para o conjunto de cânceres não foi constante, com queda em homens entre 1980 e 1990, seguida de um aumento entre 1990 e 1999 que continuou de forma menos pronunciada até 2017. Entre mulheres ocorreu ligeira queda entre 1980 e 1989, seguida de aumento até 1996 e queda até 2004, quando as taxas voltaram a subir.

Dados de outros países, em sua maioria desenvolvidos, apontam para a queda marcante da mortalidade por câncer alcançada nas duas últimas décadas^3^^,^^17^, atribuída às intervenções preventivas, à introdução de rastreamento e, principalmente, aos avanços em procedimentos diagnósticos e terapêuticos^18^. No entanto, os avanços da medicina nem sempre são acessíveis para todos os indivíduos, principalmente em contextos de desigualdade socioeconômica^19^.

A maior queda detectada no período estudado ocorreu para o câncer de estômago em todas as regiões, com exceção da região Norte como um todo e do interior da região Nordeste. O declínio, já relatado em estudos anteriores^5^, pode ser explicado por uma menor exposição a fatores reconhecidamente cancerígenos como a ingestão de alimentos salgados e melhor preservação dos alimentos^20^. Há, contudo, necessidade de melhor compreender o aumento constatado entre os residentes da região Norte e dos municípios fora das capitais nas regiões Nordeste.

O câncer de colo do útero declinou em todas as regiões, mas não entre mulheres do interior da região Norte, onde as taxas em 2017 chegam a ser três vezes mais frequentes do que na região Sudeste. Algum efeito do rastreamento com base no exame de Papanicolau, introduzido em todo o país a partir de 1992^21^, foi capaz de reverter, em parte, a tendência de crescimento das taxas, porém ainda existem mulheres sem acesso ao rastreamento no interior das regiões Norte e Nordeste, fato já relatado anteriormente com dados até 2011^11^.

A correlação inversa entre o índice de desenvolvimento humano (IDH) e as estimativas nacionais de câncer do colo do útero é bem relatada na literatura^4^. A razão de mortalidade por câncer do colo do útero nos EUA entre localidades mais pobres e mais ricas chegou a 2 entre 2012 e 2016^22^. Vários indicadores podem exemplificar a desigualdade na mortalidade por esse tipo de câncer entre as regiões brasileiras, por exemplo, o número de anos potenciais de vida saudável perdidos aos 60 anos de idade é duas vezes mais alto na região Norte em comparação à região Sudeste^23^.

A região Sul é a que apresenta o maior índice de desenvolvimento humano municipal (IDHM) do país (0,766), ao passo que as regiões Norte e Nordeste, apesar de terem tido crescimento mais acelerado entre 2000 e 2010, têm os mais baixos^24^. Embora tenha havido melhora nos indicadores de saúde no Brasil entre 1990 e 2016, a carga de doença continua maior nos estados das regiões Norte e Nordeste em comparação com as regiões Sudeste e Sul^25^.

A experiência de países de alta renda, como os da América do Norte, de parte da Europa e o Japão, são um exemplo de que o investimento em programas efetivos de rastreamento fizeram com que hoje o câncer do colo do útero seja considerado uma doença rara^26^. A prevenção secundária com o uso de citologia oncótica, no entanto, pressupõe a operação estruturada de uma rede de atenção com qualidade laboratorial. Somado a isso, o efeito da vacina contra o papilomavírus humano (HPV) terá efeitos no longo prazo e não substitui o rastreamento para a doença. Nesse sentido, a introdução do teste de detecção de HPV pode acelerar a efetividade do rastreamento^27^ e deve ser uma opção a se considerar em países como o Brasil, que ainda convive com altas taxas da doença.

As mudanças demográficas no Brasil, com progressivo envelhecimento populacional, aumento da expectativa de vida e redução da fecundidade, somadas ao aumento da obesidade^28^, têm como consequência o aumento da incidência do câncer de mama. Embora a incidência do câncer de mama no Brasil seja menor do que nos EUA e em outros países da Europa, a mortalidade por esse câncer é maior em todos os grupos etários, por consequência da alta prevalência de casos diagnosticados em estágio avançado^29^. No entanto, mesmo com tendência a aumento da incidência, as taxas de mortalidade passaram a declinantes nas capitais da região Sul de 1990 a 2017 e Sudeste de 1996 a 2017, o que pode ser efeito de melhor acesso ao diagnóstico e tratamento de mulheres residentes nessas capitais^11^.

Essa mesma tendência de diminuição nas regiões mais desenvolvidas do Brasil acontece em relação ao câncer de próstata, para o qual se percebe queda entre os homens das capitais desde 1997 e mais tardiamente entre os residentes do interior. Tal tendência pode ter relação com as possibilidades de oferta de serviços especializados para o tratamento dos casos diagnosticados precocemente^30^.

O aumento da incidência do câncer de próstata nos países desenvolvidos parece estar relacionado ao aumento de acesso aos serviços de saúde, assim como ao aumento do registro de casos^31^. Foi observada elevação independente da adoção do teste de detecção do antígeno prostático específico (PSA) de rotina, o que sugere a interferência do estilo de vida ocidental, que leva a aumento da obesidade e inatividade física^32^. Embora não estejam claras as razões pelas quais as taxas de mortalidade vêm caindo na maioria dos países ocidentais, essa queda tem sido atribuída à detecção precoce e a melhora no tratamento. A introdução do teste de PSA pode ter influenciado a detecção precoce e com isso reduzido a mortalidade. No entanto, o sobrerrastreamento, que pode levar a efeitos indesejáveis do tratamento, contraindica o uso desse teste como programa de rastreamento^31^. O fato de terem sido observadas as mais altas APC das taxas de mortalidade por câncer de próstata nas regiões Norte e Nordeste pode sugerir estar em curso nelas uma rápida absorção do estilo de vida sedentário coexistindo com uma baixa capacidade de oferta de serviços especializados para diagnóstico e tratamento^30^.

O câncer de pulmão foi declinante entre homens das regiões Sudeste e Sul e nos residentes das capitais da região Centro-Oeste. Isso, contudo, não se verificou entre as mulheres, para as quais, de forma generalizada, as taxas foram ascendentes no período estudado. Nesse caso, por se tratar de um câncer com letalidade ainda alta, a diminuição da mortalidade se dá em função da diminuição da incidência. O aumento da mortalidade por câncer de pulmão entre mulheres já havia sido identificado em estudos em períodos anteriores^33^^,^^34^.

A prevalência de fumantes no Brasil diminuiu em 19% entre 2008 e 2013, com declínio em todas as regiões, na área urbana e na rural, e na maioria dos estados. Houve queda em todas as faixas de escolaridade, porém as mais altas prevalências foram vistas entre pretos e pardos^35^. O tabagismo no Brasil é um dos mais baixos do mundo e isso poderá ter impacto positivo no futuro, desde que as políticas de controle, regulação e prevenção sejam mantidas e fortalecidas^36^. Embora a redução da prevalência de fumantes tenha ocorrido de forma similar nos dois sexos, deve ser considerado que a introdução do tabagismo entre mulheres foi mais tardia; portanto, o aumento da ocorrência do câncer de pulmão ainda reflete essa condição^36^.

O câncer colorretal, terceiro e quarto mais frequente entre mulheres e homens, respectivamente, mostra um padrão evolutivo que merece atenção. Embora as taxas nas regiões Sudeste e Sul sejam maiores entre homens, elas são três vezes superiores às da região Norte.

A ocorrência de câncer colorretal mostra padrões diferenciados entre países. Em vários países do Leste Europeu, da América Latina e da Ásia, observou-se tendência ascendente na incidência e na mortalidade. Já no Canadá, em países da Europa e em Singapura, a incidência continua aumentando, enquanto há declínio na mortalidade. No grupo restrito a países de alto IDH, há tendência de queda tanto na incidência como na mortalidade^35^.

Chama atenção o fato de que, nas regiões Norte e Nordeste, as tendências da mortalidade por câncer colorretal tiveram aumento mais expressivo no interior, porém não se observou diferença de magnitude entre os sexos. Isso pode ser um indicativo de que o aumento da obesidade e do sedentarismo, fatores de risco clássicos para este tipo de tumores^37^, esteja ocorrendo de forma generalizada no país. Esse aumento pode ser consequente a um aumento real na incidência, que, somado à falta de acesso ao diagnóstico e tratamento especializado, pode ter efeito sobre a mortalidade. Grandes diferenças no acesso a serviços de saúde são vistas entre as regiões no Brasil, havendo uma maior proporção de consultas médicas nas regiões Sul e Sudeste, entre pessoas com melhores condições de vida e em regiões com maiores IDH^30^.

A principal limitação deste estudo decorre da qualidade das informações sobre os óbitos no país. Devido à grande discrepância em termos de completitude da informação sobre a causa básica do óbito, principalmente nas regiões Norte e Nordeste nas duas primeiras décadas do período estudado^12^^,^^13^, foi feita correção dos óbitos maldefinidos e daqueles registrados como câncer de útero sem outra especificação. Foram assim distribuídos todos os óbitos registrados como causa básica maldefinida que são classificados no capítulo XVI da CID-9 de 1979 a 1995 e no capítulo correspondente da CID-10 (XVIII) a partir de 1996.

Alguns estudos prévios^6^^,^^38^^,^^39^ seguem a metodologia proposta pelo estudo Carga Global de Doença (Global Burden of Disease – GBD)^40^, que redistribui os óbitos registrados entre causas consideradas como códigos *garbage*. Nesse conjunto de códigos, são incluídos, além dos óbitos registrados nos capítulos XVI da CID-9 e XVIII da CID-10, outras causas maldefinidas e diagnósticos incompletos vindos de outros capítulos. Optou-se no presente estudo por não incluir os demais códigos inespecíficos fora dos capítulos XVI da CID-9 e XVIII da CID-10 pela ausência de estudos que validem a contribuição da redistribuição dos óbitos registrados nos demais códigos inespecíficos entre os tipos específicos de câncer^41^. Essa opção pode ter subestimado ligeiramente a magnitude das taxas, mas não prejudicou a comparação entre as regiões no período.

Estudos anteriores que mostraram a tendência de câncer no Brasil, com metodologia aplicando correção dos dados de óbito, incluíram períodos menores do que aqui apresentados: 1980–2006^5^, 1990–2015^6^ e 1996–2016^39^. No caso do câncer, é importante entender a evolução da incidência e da mortalidade por um período maior, como foi possível mostrar com dados do SIM desde 1978. Para otimizar a correção dos dados de óbito, ela foi feita de forma desagregada segundo local de residência, faixa etária, sexo e ano-calendário. Por esse motivo, as tendências aqui apresentadas diferem daquelas que não efetuam correção dos dados do SIM, mas estão mais próximas de detectar as direções e mudanças de tendência na mortalidade por câncer. Deve ser enfatizado que, nas duas classificações da CID, os códigos para o total de neoplasias malignas e para os tipos específicos selecionados, bem como os códigos referentes à causa básica maldefinida, são totalmente correspondentes, o que permitiu analisar a tendência em todo o período com segurança de que a utilização das duas respectivas versões não introduziu viés de classificação^42^.

Um outro aspecto a ser considerado é a cobertura do registro de óbitos. Embora tenha melhorado sensivelmente no país ao longo dessas quatro décadas, o sub-registro ainda existe em determinadas áreas, especialmente nos estados das regiões Norte e Nordeste^43^^,^^44^. Como este estudo analisou dados desde 1978 até 2017, optou-se por não realizar a correção do sub-registro por não serem encontrados estudos que avaliam a cobertura do SIM a partir de 2013. Essa opção pode ter subestimado, em parte, as taxas calculadas para as regiões Norte e Nordeste. No entanto, como os principais tipos de câncer ocorrem a partir da idade adulta, e considerando que a maior parte do sub-registro acontece na faixa etária de até um ano de idade^45^, a subestimação em função do sub-registro não deve ter sido tão alta.

Quanto à aplicação da regressão linear por partes para avaliar a tendência no tempo, deve ser considerado que, se o intervalo de tempo for muito pequeno, mudanças de trajetória podem não apresentar significância estatística em função do número reduzido de pontos. No entanto, isso não ocorreu pois, em se tratando de um longo período de estudo, as APC que foram calculadas cobriram períodos de no mínimo quatro anos. Além disso, os resultados deste estudo consideraram apenas os intervalos que alcançaram significância estatística. Deve ser considerado também que o número de óbitos em cada unidade de análise não foi pequeno, considerando que os dados foram desagregadas até o nível das capitais e do interior de cada grande região, o que diminuiu a possibilidade de instabilidade na estimativa das taxas e de haver alta heterogeneidade na distribuição delas, ou seja, menor variância.

Outra limitação dos modelos lineares para a análise de tendência é a possibilidade de autocorrelação residual. Neste trabalho, os modelos com diagnóstico de autocorrelação nos resíduos foram reestimados via mínimos quadrados generalizados com uma estrutura de correlação autorregressiva de primeira ordem – AR(1). Não foram identificadas estruturas de correlação de ordem mais elevadas. Logo, quando existente, a autocorrelação foi adequadamente tratada no modelo.

Vale enfatizar que, ao mesmo tempo que se assiste à redução de alguns cânceres relacionados à pobreza, é real o aumento de novos casos de câncer associados a fatores decorrentes da história reprodutiva, hormonal e da dieta^4^. Assim, por exemplo, o ponto de cruzamento no tempo entre as curvas de incidência do câncer do colo do útero e o de mama constitui-se como um indicador típico dos padrões de transição de câncer em países de média e baixa renda^46^.

Foi visto que, nas regiões Sudeste e Sul, esse ponto de cruzamento entre a mortalidade por câncer de mama e câncer do colo do útero ocorreu antes da década de 1980 entre as mulheres residentes nas capitais. Já nessas mesmas regiões, para as residentes no interior, essa transição se deu mais tarde. Situações intermediárias ocorreram nas regiões Nordeste e Centro-Oeste, onde o ponto de cruzamento da mortalidade entre estes dois tipos de câncer nas capitais aconteceu em 1992 e 1993, respectivamente. No interior, porém, ele se deu apenas em 2008 na região Nordeste e em 2007 na região Centro-Oeste. A situação mais extrema é vista na região Norte, onde essa transição ainda não ocorreu nem nas capitais, nem no interior, indicando um quadro encontrado em países pobres^4^.

Na mesma lógica, entre os homens podem ser identificados os pontos de cruzamento das curvas de mortalidade por câncer colorretal, que se associa positivamente com IDH^35^, e o de estômago, que é conhecidamente relacionado à pobreza^4^. Foi nas capitais da região Sul que esse cruzamento se deu primeiro; no ano de 1998, as taxas de óbito por câncer colorretal superaram as de estômago. Nessa mesma região, para os que residem no interior, isso só veio a acontecer em 2016. Na região Sudeste essa transição ocorreu em 2007 nas capitais e apenas em 2016 no interior. Nas regiões Centro-Oeste e Nordeste, apenas nas capitais as taxas de mortalidade por câncer colorretal em homens superaram as de estômago (2014 e 2017, respectivamente). O padrão de maior carência foi visto na região Norte, onde as taxas de câncer de estômago são mais altas do que as de colorretal, tanto nas capitais quanto no interior.

Alguns resultados encontrados demandam um exame cuidadoso para entender os mecanismos implicados tanto em relação à acurácia da informação de óbitos como na qualidade da assistência à saúde. O crescimento marcado dos cânceres de próstata, colorretal e de mama no interior das regiões Norte e Nordeste merece investigação detalhada. Uma hipótese seria estar ocorrendo uma melhora da capacidade diagnóstica, porém com proporção alta de casos detectados em estágios tardios^29^. A hipótese de melhora da informação sobre causa básica também não pode ser afastada, mesmo considerando que foi efetuada a correção com redistribuição dos óbitos malclassificados. É possível que nessas regiões a correção não tenha sido suficiente para se aproximar do peso real da ocorrência desses tumores. Destaque também deve ser dado ao crescimento generalizado do câncer de pulmão em mulheres, o que foi altamente expressivo no interior das regiões Norte e Nordeste.

Por fim, é preocupante o crescimento das taxas para o conjunto dos cânceres em anos recentes, em especial no interior das regiões Norte e Nordeste. Isso pode significar um aumento real na incidência devido a uma maior exposição a fatores de risco relacionados a um estilo de vida sedentário, mas, em se tratando de mortalidade, as dificuldades de acesso, em especial à detecção precoce e ao tratamento de tumores de bom prognóstico como os de mama e de próstata, podem levar a um aumento da mortalidade.

O Brasil é um país em transição econômica e, durante essas quatro décadas, passou por várias modificações demográficas, sociais e políticas^47^, que interferem na tendência dos tipos específicos de câncer, configurando um perfil específico de transição que assume características próprias com contrastes regionais importantes. As quedas dos níveis de mortalidade, natalidade e fecundidade verificadas a partir de 1950 indicam que várias modificações não se deram de forma uniforme e simultânea, mas marcadamente formatada pela desigualdade socioeconômica entre regiões^23^ e dentro delas. Nas regiões Sudeste, Sul e Centro-Oeste, esse processo foi mais acelerado, enquanto nas regiões Norte e Nordeste os níveis de mortalidade e fecundidade foram mais elevados, com estruturas etárias menos envelhecidas^47^.

A partir da criação do Sistema Único de Saúde, incorporado à Constituição Federal em 1988, houve uma grande expansão dos serviços públicos, com significante melhora na equidade em saúde, porém não suficiente para diminuir as extremas desigualdades em todo o país^48^. Em anos recentes, em função de sucessivas crises econômicas e políticas, alguns indicadores sociais já começam a apontar retrocessos, e um cenário de incerteza se configura para os próximos anos. Estudo recente, por exemplo, concluiu que o aumento do desemprego no país entre 2012 e 2017 levou a um excesso de 30.000 mortes, principalmente por câncer e doenças cardiovasculares^49^. As mudanças demográficas, as desigualdades socioeconômicas e as crises políticas que se sucederam no país ao longo de quatro décadas podem explicar, em parte, a diminuição da mortalidade nas regiões Sudeste e Sul e o aumento entre residentes dos municípios fora das capitais, em especial nas regiões Norte e Nordeste.

Os resultados encontrados refletem a existência de padrões distintos de magnitude e tendência de tipos específicos de câncer que por vezes são contrastantes e podem refletir processos de adoecimento e morte com dinâmicas particulares entre as grandes regiões do país e entre residentes de capitais e demais municípios. Ao mesmo tempo que para moradores das capitais das regiões mais desenvolvidas há uma tendência à diminuição da mortalidade, nas regiões mais pobres os aumentos expressivos, sobretudo no interior, deixam claro um perfil de extrema iniquidade em saúde.
